# Advances in Surface/Interface Engineering of Under-Water Superaerophobic Electrodes for Hydrogen Evolution Reaction by Manipulating of Bubbles

**DOI:** 10.1007/s40820-026-02198-5

**Published:** 2026-05-04

**Authors:** Annan He, Fengxiang Chen, Jun He, Xian Zhang, Shangzhen Xie, Na Yao, Zhiguang Guo, Weilin Xu

**Affiliations:** 1 Ministry of Education Key Laboratory for the Green Preparation and Application of Functional Materials, Hubei Key Laboratory of Polymer Materials, Hubei University, Wuhan, 430062 People’s Republic of China; 2 State Key Laboratory of New Textile Materials and Advanced Processing, Wuhan Textile University, Wuhan, 430200 People’s Republic of China; 3 State Key Laboratory of Silicate Materials for Architectures and School of Materials Science and Engineering, Wuhan University of Technology, Wuhan, 430070 People’s Republic of China

**Keywords:** Hydrogen evolution reaction, Superaerophobic electrodes, Micro/nanostructure, Bubble manipulation, Electrocatalysis

## Abstract

The ever-increasing consumption of fossil fuels has led to environmental crises, which accelerated the quest for sustainable hydrogen energy. Among various production routes, water electrolysis stands out as a promising approach. However, the efficiency of hydrogen evolution reaction is limited by the adhesion of gas bubbles on electrode surfaces, which blocks active sites, increases overpotential, and limits mass transfer. This review highlights the design of micro/nanostructured array electrodes to achieve underwater superaerophobicity, reducing bubble adhesion, facilitating the nucleation and rapid release of ultrasmall bubbles, thereby contributing to reduce in overpotential, faster bubble growth, enhanced mass transport, and improved catalyst stability. We summarize recent advances in fabrication strategies of such electrodes, focusing on micro/nanostructural designs, covering from 0 to 3-dimensional structures. Additionally, the role of hydrophilic gels in optimizing superaerophobicity is discussed. Finally, challenges and future directions are addressed, including bubble dynamics accurate modeling, development of high activity and stability catalysts, intelligent adaptive electrode structure and active bubble regulation, and the integration of artificial intelligence and deep learning for guided electrode design. This review aims to provide a comprehensive perspective on how superaerophobic electrode design address bottlenecks in gas-evolving electrodes, paving the way toward more efficient and economical hydrogen production.

## Highlights

- Comprehensive mechanistic understanding of bubble nucleation, growth, and detachment on superaerophobic electrodes.
- Detailed explanation of the mechanism by which superaerophobic electrodes improve the efficiency of hydrogen evolution reaction.
- Comprehensive analysis of micro/nanostructures and hydrophilic gels in bubble manipulating strategies during hydrogen evolution reaction.

## Graphical abstract

**Figure:**
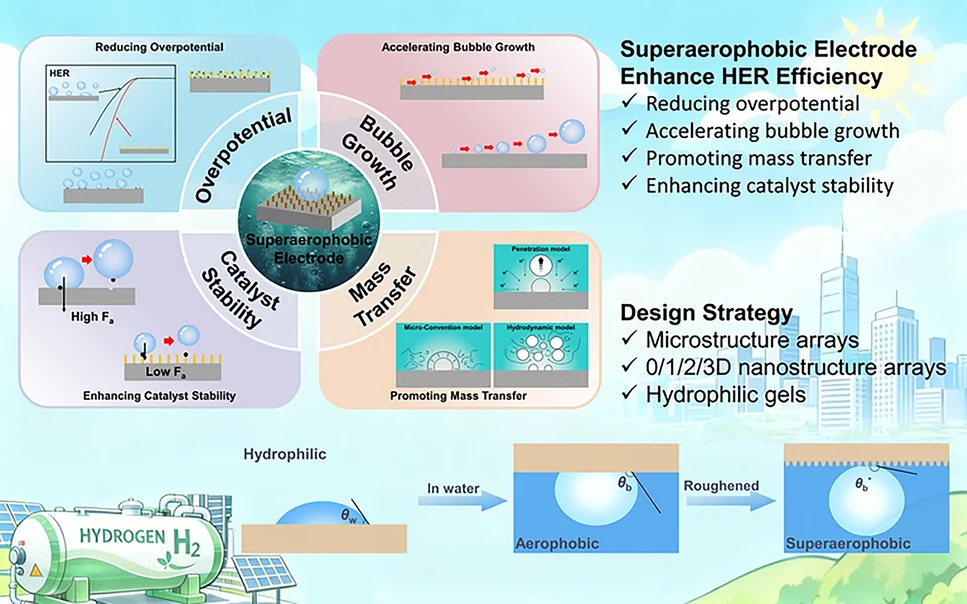

## Introduction

As environmental pollution becomes increasingly severe due to the increased consumption of fossil fuels, seeking for clean and renewable energy has received more and more attention. Hydrogen (H_2_) has become an ideal energy carrier due to its small molecular weight, high energy density (142 MJ kg^−1^ for higher heating value [1], and ~ 120 MJ kg^−1^ for lower heating value [2]) and environmentally friendly products (only water) [3]. At present, the production of hydrogen in industry mainly includes steam methane reforming, coal gasification, cracking of petroleum, water electrolysis and so on [4]. The first three account for the majority of industrial hydrogen production but all produce carbon pollution, while green water electrolysis hydrogen production accounts for only ~ 0.1% of global hydrogen production due to the low technological maturity.

In addition to the development of more stable, efficient and renewable catalysts, another major limitation to the development of hydrogen production technology by water electrolysis is the common problem for gas evolution reaction (GER), that is, in the process of generating bubbles, the bubbles adhering to the electrode will block the contact between the electrode and the electrolyte. Only after the bubbles are detached from the electrode can they reconnect to form a pathway and continue catalysis. This causes the GER on the electrode to be in a state of continuous start and stop. If the bubbles are quickly detached from the electrode when they grow to a very small size, this will not only increase the reaction efficiency, but also reduce the energy loss during gas evolution, which can greatly promote the development of hydrogen production technology by water electrolysis. Therefore, it is of great significance in the hydrogen evolution industry and even the entire industrial sector to deeply exploring the dynamics of bubbles on solid surfaces and designing superaerophobic electrodes to achieve reliable control of bubbles [5, 6].

The development of the hydrogen evolution reaction (HER) has evolved through three key stages: embryonic, industrial commercialization, and efficient clean production. It began in 1789 with the decomposition of water using an electrostatic generator by Paets van Troostwijk and Deimann [7, 8], followed by Michael Faraday’s principles of electrolysis in 1834 [9] and Dmitry Lachinov’s alkaline water electrolysis patent in 1888 [10]. The twentieth century saw industrialization, with advancements like unipolar electrolyzer invented by German researchers Garuti and Schuc Kert in the early 1900s, while Schmidt-Oerikon introduced the industrial bipolar electrolyzer [11], followed by high-pressure electrolyzer developed Ewald A. Zdansky of Lonza in 1948, and then proton exchange membrane (PEM) hydrogen generator, respectively, designed by DuPont and General Electric [12]. However, progress slowed due to cheaper alternatives like steam methane reforming. In the twenty-first century, the focus has shifted back to water electrolysis, driven by the global push for sustainable and green energy solutions.

Interfacial wettability science has a parallel history, beginning in 1805 with Thomas Young’s introduction of the contact angle concept [13]. The Marangoni effect, studied in 1865 identified interfacial tension-driven flow arising from surface tension gradients [14]. Subsequent theoretical advances including Wenzel’s roughness theory in 1936 [15], Cassie and Baxter’s 1944 composite wetting model provided a framework for understanding liquid behavior on rough and porous surfaces [16]. The twenty-first century brought breakthroughs, Jiang’s “gecko” [17] and “lotus” [18] models in 2005, which described superhydrophobic surfaces with hierarchical micro/nanostructures, and his 2014 work on superwetting surfaces in air/water/oil systems, paving the way for applications in oil–water separation, anti-fouling coatings, and especially GER [19]. Current research tends to focus on the Marangoni convection at the bubble interface induced by gradients in local temperature, concentration, or surface-active substances around bubbles during the GER, which redistributes dissolved gases and electrolytes, thereby affecting the bubble growth dynamics, detachment behavior, and mass transfer near the electrode surface [20].

Since Jiang’s work on the wettability of air/water system on solid surface has driven the development of superaerophobic electrodes for HER in 2014, by leveraging hierarchical surface architectures, such as “Wenzel’s,” “Cassie’s,” “Lotus” and “Gecko” states, researchers designed electrodes that minimize bubble adhesion and enhance mass transfer. Recent advancements focus on scalable fabrication, faster bubble release, and stronger mass transfer capabilities, making these electrodes ideal for large-scale hydrogen production.

Considering the importance and urgency of bubble manipulation in HER, we briefly describe the reaction and catalytic mechanism of HER, overview the necessity and strategies of constructing superaerophobic electrodes and discuss recent advances in this field. At last, focusing on the strategies for constructing advanced micro/nanostructures for superaerophobic electrodes, we propose challenges and solutions associated with these strategies aiming to promote further exploration of this field by relevant researchers and practitioners.

## Mechanism of HER

Water splitting is a process in which a reduction reaction occurs at the cathode to produce H_2_ and an oxidation reaction occurs at the anode in an energized aqueous electrolyte, the reaction is shown as follows Eq. 1 [21]:

1 $$2 {H}_{2}O \to 2 {H}_{2} \uparrow + {O}_{2} \uparrow$$

It is worth noting that the HER occurs at the cathode, and the reaction mechanism is different in various pH values or solutes. Simply put, it is the reduction of H_2_O in alkaline or neutral conditions or the reduction of H^+^ in acidic conditions to produce H_2_. There are two pathways for both of the reactions, the Volmer-Heyrovsky reaction and the Volmer-Tafel reaction [22]. Here, the reaction mechanism and products of HER in alkaline, neutral and acidic conditions will be discussed.

### In Alkaline or Neutral Condition

**Fig. 1:**
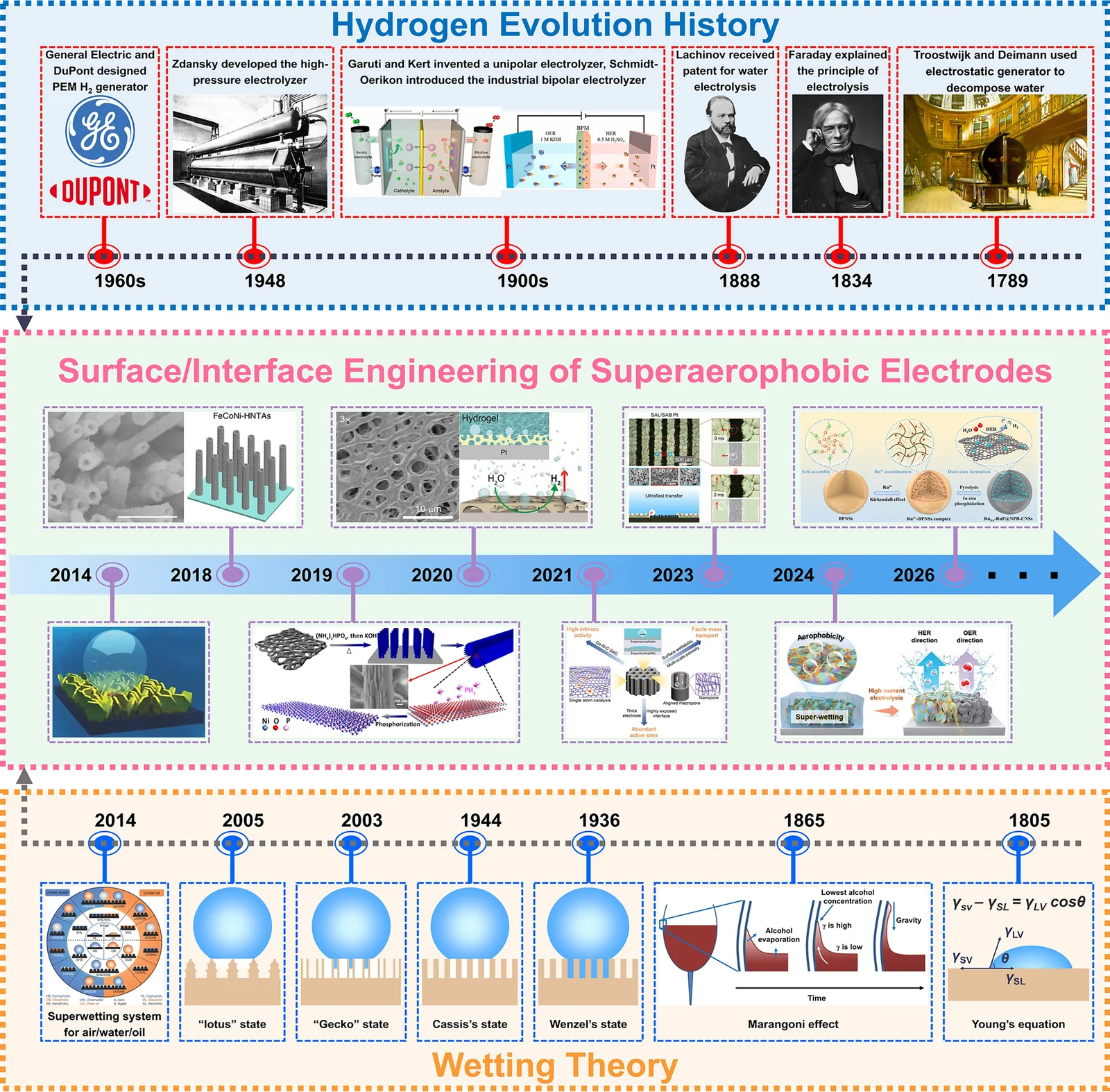
The development history of superaerophobic electrodes for HER

In alkaline or neutral conditions, the donor of H atoms is mainly obtained from the decomposition of H_2_O molecules because of the low concentration of H^+^ in the medium (Fig. 1). The first step is the Volmer step: a H_2_O molecule combines with an electron (e^−^) to form OH^−^ and a H atom, which is adsorbed on the active site (AS) of the electrode to form AS-H^*^ (Eq. 2). Then the Heyrovsky step or the Tafel step occurs according to the coverage of AS-H^*^ to produce H_2_ [23]. Specifically, the Heyrovsky step is that under a low AS-H^*^ coverage, the adsorbed H atom (i.e., AS-H^*^) further binds to a H_2_O molecule and an e^−^ to undergo electrochemical desorption to form a H_2_ molecule and OH^−^ (Eq. 3), while the Tafel step occurs when the AS-H^*^ coverage is high, two AS-H^*^ combine and undergo chemical desorption to produce a H_2_ molecule (Eq. 4) [24, 25]:

2 $${\mathrm{Volmer}}\,{\mathrm{Step}}:{\mathrm{H}}_{{2}} {\mathrm{O}} + e^{ - } + {\mathrm{AS}} \to {\mathrm{OH}}^{ - } + {\mathrm{AS}} - {\mathrm{H}}^{*}$$

3 $${\mathrm{Heyrovsky}}\,{\text{Step: AS - H}}^{*} {\text{ + H}}_{{2}} {\text{O + }}e^{ - } { } \to {\text{ H}}_{{2}} {\text{ + OH}}^{ - } {\text{ + AS}}$$

4 $${\mathrm{Tafel}}\,{\text{Step: 2 AS - H}}^{*} { } \to {\text{ H}}_{{2}} {\text{ + 2 AS}}$$

**Fig. 2:**
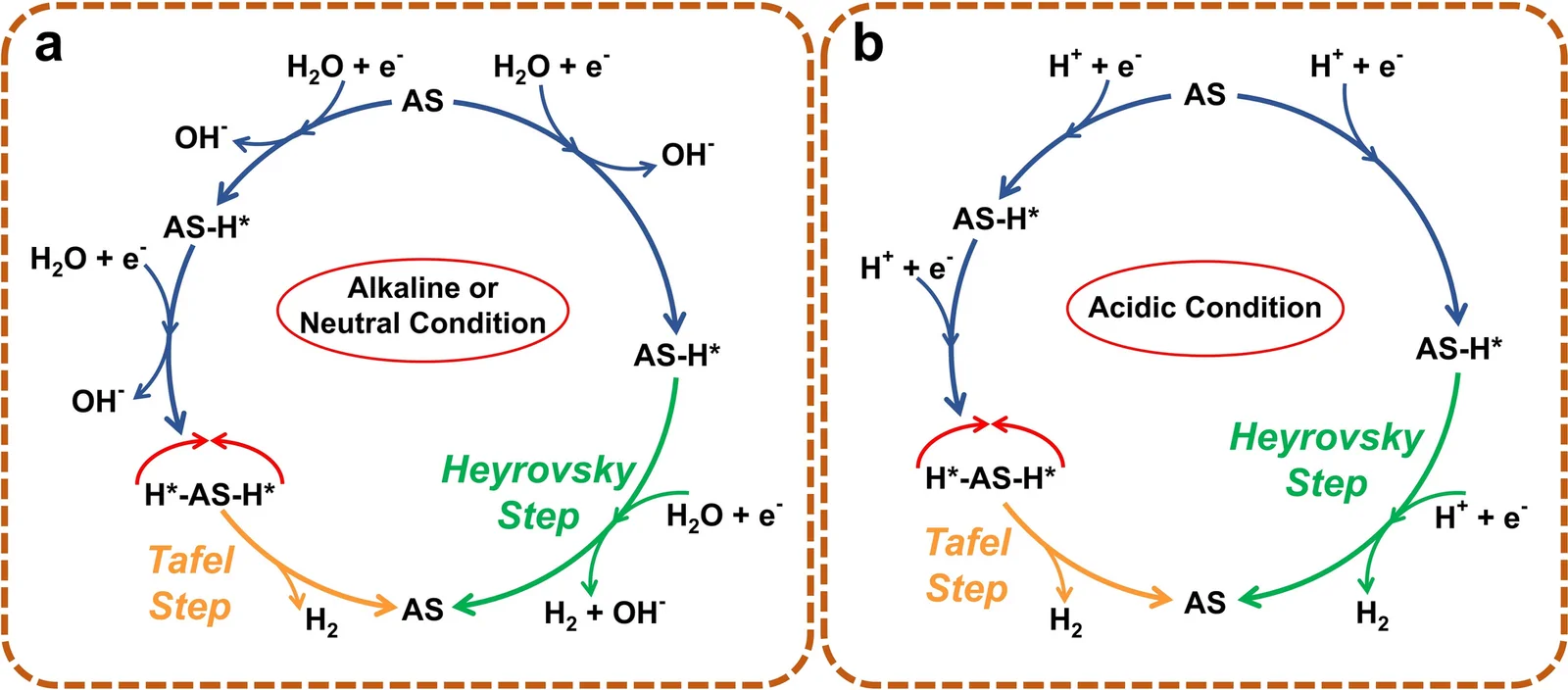
Mechanism of electrodes under different conditions for HER. Schematic of the HER mechanism at the cathode under alkaline or neutral (**a**) and acidic (**b**) conditions

The schematic is shown in Fig. 2a.

### In Acidic Condition

Similar to the reaction in alkaline or neutral condition, the HER in acidic condition also includes two key steps. The difference is that the H atoms are taken directly from the H^+^ of the medium, due to the high concentration of H^+^ in the medium. Specifically, Volmer step is that a H^+^ binds to an e^−^ and is absorbed to the AS of the electrode, resulting in AS-H^*^ (Eq. 5). The Heyrovsky step undergoes when the AS-H^*^ coverage is low, then the AS-H^*^ is coupled with a H^+^ and an e^−^ to produce a H_2_ molecule by electrochemical desorption (Eq. 6). While the Tafel step occurs when two AS-H^*^ combine and undergo chemical desorption to produce a H_2_ molecule when the AS-H^*^ coverage is high (Eq. 7, same to Eq. 4) [24, 25]:

5 $${\mathrm{Volmer}}\,{\mathrm{Step}}: {\mathrm{H}}^{ + } + e^{ - } + {\mathrm{AS}} \to {\mathrm{AS}} - {\mathrm{H}}^{*}$$

6 $${\mathrm{Heyrovsky}}\,{\text{Step: AS - H}}^{*} {\text{ + H}}^{ + } {\text{ + e}}^{ - } { } \to {\text{ H}}_{{2}} {\text{ + AS}}$$

7 $${\mathrm{Tafel}}\,{\text{Step: 2 AS - H}}^{*} { } \to {\text{ H}}_{{2}} {\text{ + 2 AS}}$$

The schematic is shown in Fig. 2b.

## Effect of Superaerophobic Electrodes on GER Efficiency

**Fig. 3:**
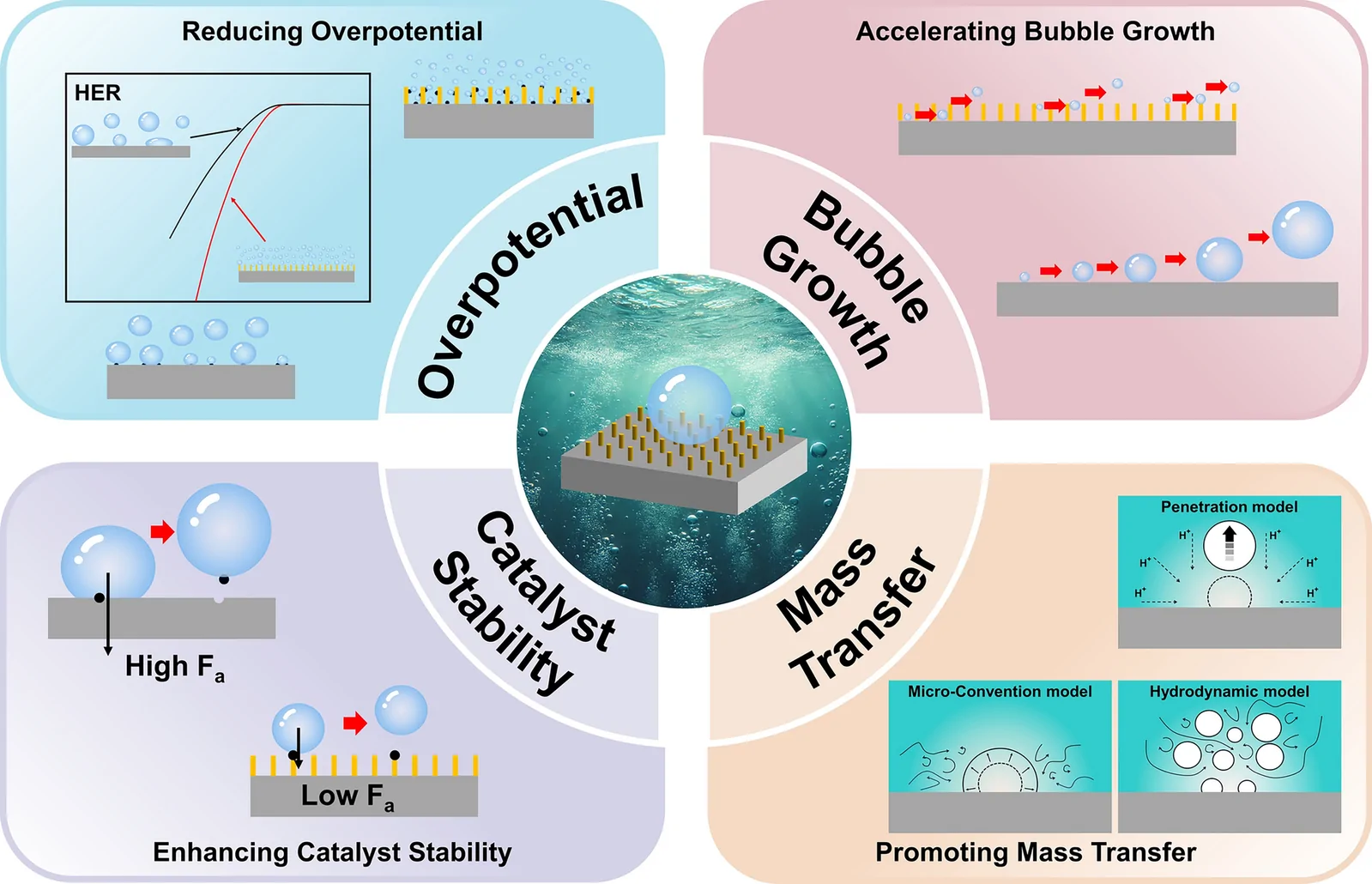
Overview of the mechanisms on superaerophobic electrodes for improving GER efficiency (where *F*_a_ is adhesion force of bubbles to catalyst)

During the GER process, bubbles typically nucleate in the cavities or defects of nano-scale electrodes and grow by absorbing supersaturated gas molecules from the surrounding [26]. In the context of superaerophobic electrodes, the reaction efficiency can be significantly enhanced through multiple mechanisms, particularly demonstrating remarkable advantages in reducing overpotential, accelerating bubble growth, and promoting mass transfer [27]. In addition, by improving the interaction between bubbles and the electrode surface, superaerophobic electrodes achieve more efficient gas generation and release, while also mitigating the adverse effects of bubbles or byproducts, thus enhancing catalyst stability [28], as we outline in Fig. 3.

### Reducing Overpotential

Superaerophobic electrodes can significantly reduce the overpotential in GER. By optimizing the micro/nanostructure of the electrode surface and reducing the interaction between bubbles and the electrode surface, superaerophobic electrodes can promote gas release more efficiently. For GERs, the overpotential (*η*) of the reaction are highly affected by bubbles at the interface of electrode and electrolyte, which can be divided into activation overpotential (*η*_act_), ohmic overpotential (*η*_ohm_), and concentration overpotential (*η*_con_). Equation is shown as follows (Eq. 8) [29, 30]:

8 $$\eta = {\eta }_{act} + {\eta }_{ohm} + {\eta }_{con}$$

**Fig. 4:**
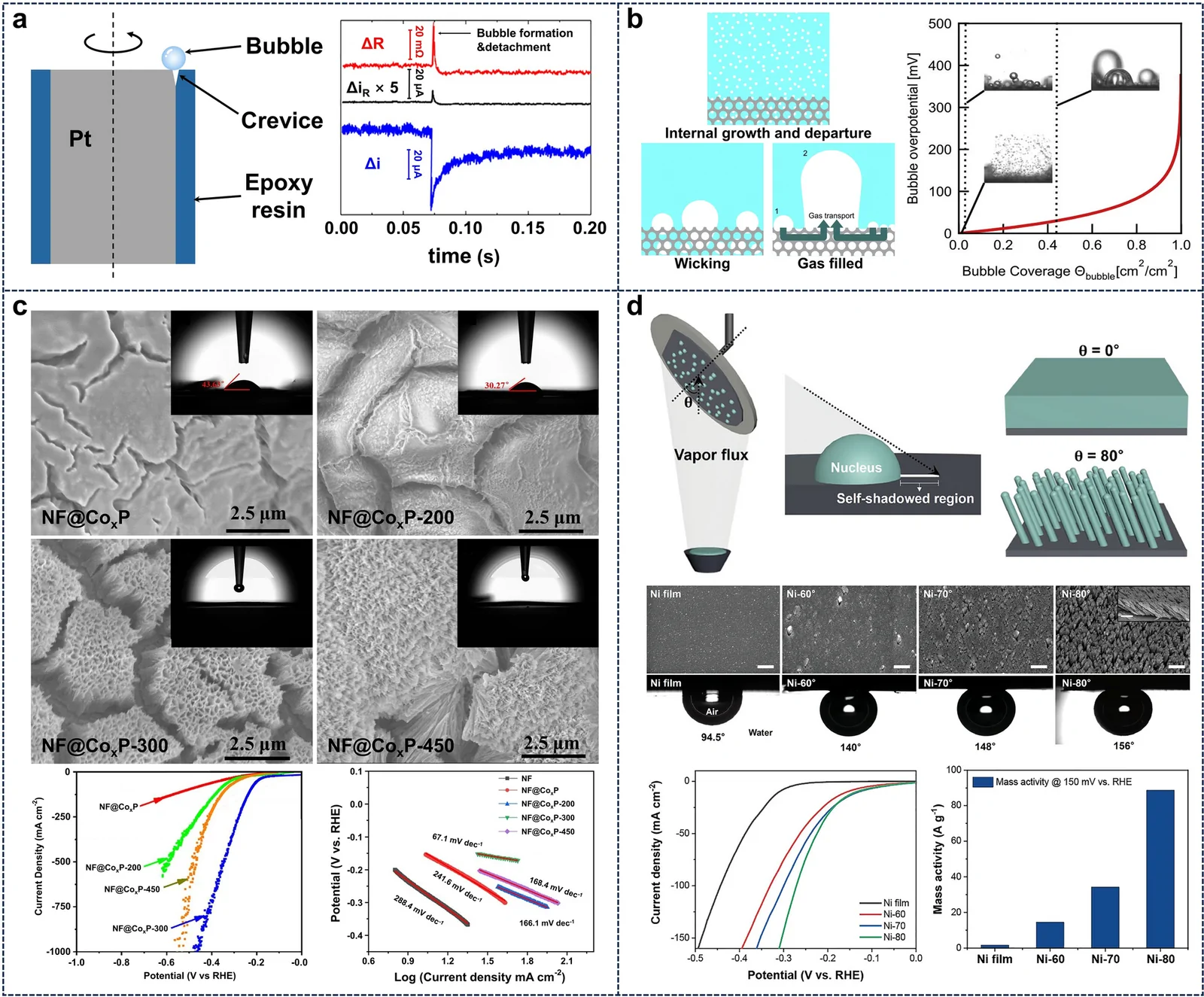
**a** Schematic diagram of the experimental design for proving the rapid detachment of bubbles can reduce the overpotential; and the corresponding changes in various electrical parameters (electrolyte resistance, Δ*R*; ohmic-current, Δ*i*_*R*_; and total current, Δ*i*) during the formation and detachment of a H_2_ bubble in HER [32]. Reproduced with permission. Copyright 2005, Elsevier. **b** Schematics of bubble growth and departure modes for electrodes with different aerophobicity; and functional curve between bubble overpotential and bubble coverage. Inset images are the actual bubble coverage for the three modes [33]. Reproduced with permission. Copyright 2021, Elsevier. **c** SEM images of Co_*x*_P catalysts with different hydrophilicities and their corresponding LSV and Tafel slope curves [34]. Reproduced with permission. Copyright 2021, Elsevier. **d** Schematic diagram of various Ni nanorod arrays prepared by oblique angle deposition, the corresponding SEM images, LSV curves, and mass activity at 150 mV vs RHE [35]. Reproduced with permission. Copyright 2023, Wiley–VCH

Among them, the activation overpotential is related to the reaction loss occurring on the electrode surface. In the GER, *η*_act_ can be obtained through Eq. 9 [27, 31]:

9 $${\eta }_{act} = a + b log(\frac{i}{{A}_{eff}})$$

where a and b are the Tafel slopes, respectively. a is related to the intrinsic catalytic activity of the electrode material, while b reflects the sensitivity of the overpotential to changes in current density. *i* is the applied macroscopic current, and *A*_eff_ is effective active area. It can be observed that for electrodes with poor hydrophilicity, the adhesion of air bubbles to the surface leads to a reduction in the *A*_eff_, which in turn results in an increase in *η*_act_. Gabrielli et al. [32] designed a hydrogen evolution device as shown in Fig. 4a. They created a crevice on a rotatable resin-coated Pt electrode as a preferential nucleation site for hydrogen bubbles. When the device is put into operation, a H_2_ bubble can be controllably and discontinuously nucleate at this crevice and detach under the shear force generated by the rotation of the electrode. Figure 4b shows the curves of electrolyte resistance (Δ*R*), ohmic-current (Δ*i*_*R*_) and total current (Δ*i*) during the formation and detachment of a H_2_ bubble. It can be found that during the formation of bubbles, Δ*R* and Δ*i*_*R*_ increase rapidly, and Δ*i* decreases rapidly. This can prove that when bubbles are attached to the electrode surface, the catalyst surface will be occupied, which will greatly increase the loss during the reaction. This is because the air bubbles covered the electrode surface, increasing the electrolyte resistance. The fact that Δ*R* and Δ*i*_*R*_ can quickly return to their initial states after the bubbles are ejected from the gap due to high-speed rotation, and that Δ*i* will also be alleviated after a period of time, effectively proves the above point. Iwata et al. [33] deposited Ni and polytetrafluoroethylene (PTFE) coating on Ni foam through the co-deposition method process, and controlled the wettability of the electrode by adjusting the coverage of the PTFE as shown in Fig. 4b. They found that as the PTFE coverage increased, the electrode gradually changed from aerophobic to aerophilic, and the bubble detachment diameter and coverage also increased significantly, causing the bubble overpotential to gradually increase from almost negligible in the initial stage to nearly 30 mV when the bubble coverage was 0.4. Therefore, reducing the bubble size can effectively reduce the coverage of bubbles on the electrode and reduce the reaction loss, thereby reducing the activation overpotential.

Chen et al. [34] altered the hydrophilicity of the Co_*x*_P catalyst by varying the treatment temperature. The hydrophilicity of the Co_*x*_P catalyst changed significantly with the temperature, reaching its optimum at 300 °C (water droplet completely wet the electrode). At this temperature, the catalytic activity and catalytic kinetics also reached their optimal levels (Fig. 4c). Kim et al. [35] prepared Ni nanorod arrays with controlled surface porosity by the oblique-angle deposition method shown in Fig. 4d. As the deposition angle increases from 60° to 80°, a distinct nanorod structure is formed on the electrode surface, and the gas-repellency gradually increases. At this point, the catalytic activity of the Ni-80° sample reaches its optimal level.

### Accelerating Bubble Growth

Superaerophobic electrodes can accelerate the formation and diffusion of bubbles by improving the bubble growth kinetics, which further improves the efficiency of the overall reaction. The bubble growth kinetics can be described by Scriven theory (Eq. 10) [36]:

10 $$R = {\beta t}^{x}$$

where *β* represents the growth coefficient and x represents the time coefficient.

**Fig. 5:**
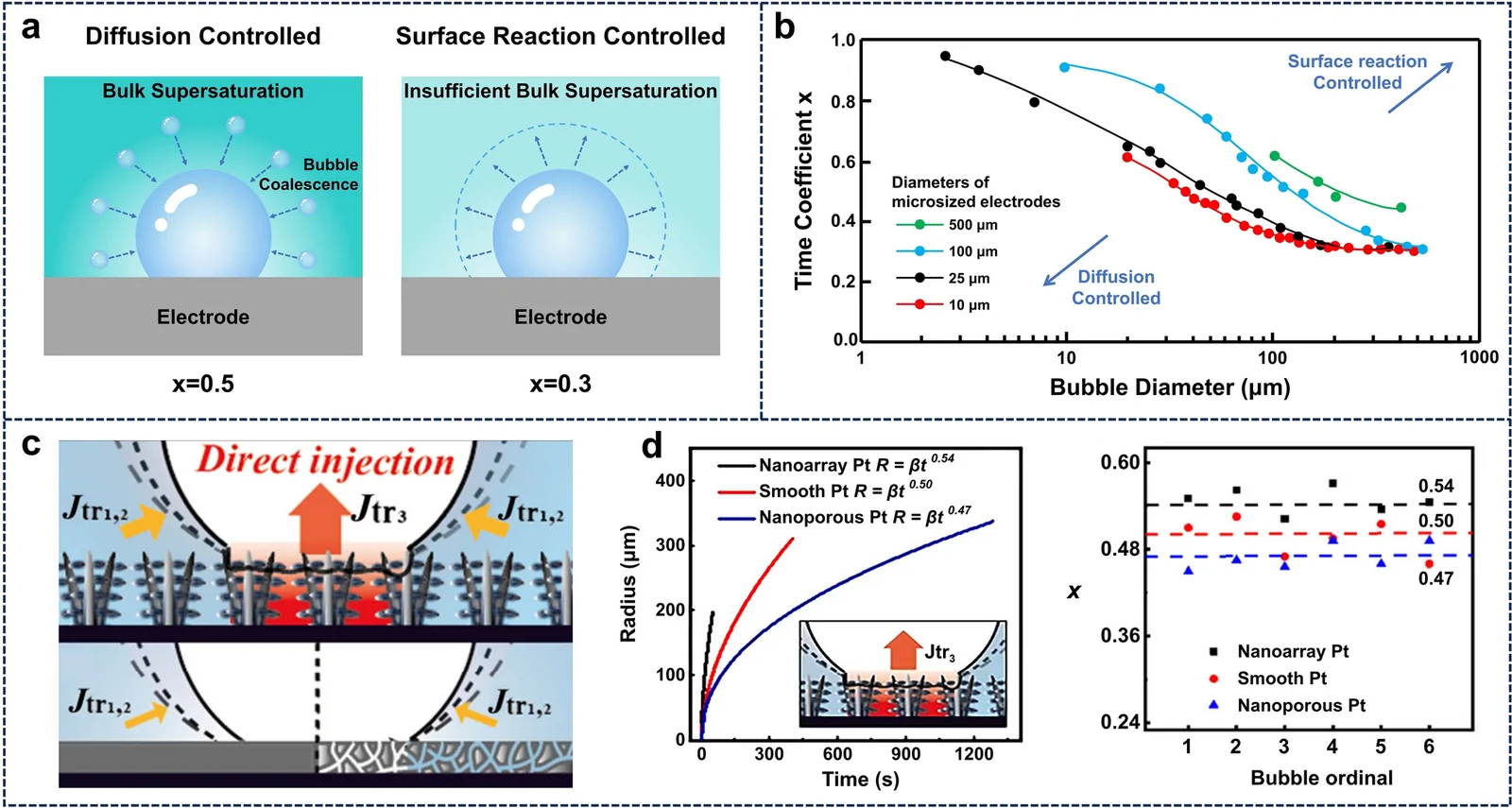
**a** Schematic illustration of the bubble growth modes on the solid surface. (left: Bubbles grow in a bulk supersaturated solution, i.e., diffusion controlled mode; right: Bubbles grow in an insufficient bulk supersaturated solution, i.e., surface reaction controlled model). **b** Influence of bubble diameter and electrode size on the time coefficient (*x*) of bubble growth kinetics [37]. Reproduced with permission. Copyright 1985, Springer Nature. **c** Schematic diagram of bubble growth mechanisms on nanoarray, smooth, and nanoporous Pt surfaces. **d** The curves of bubble diameter over time on various Pt surfaces (left), and the curves of time coefficient *x* and on various Pt surfaces (right) [38]. Reproduced with permission. Copyright 2021, Springer Nature

There are two modes for the growth of bubbles in GER: when the concentration of gas molecules dissolved in the bulk solution is higher than the solubility of the gas, i.e., supersaturated state, the gas molecules will diffuse into nearby bubbles. This is the diffusion-controlled mode, and the *x* value is ~ 0.5 (Fig. 5a, left); when the supersaturated state of the bulk solution is insufficient, the gas molecules for bubble growth are mostly provided by the surface reaction of the electrode. This is the surface reaction-controlled mode, and the *x* value is ~ 0.3 (Fig. 5a, right). Brandon and his co-worker Kelsall [37] subjected microelectrodes with different sizes to the GER and measured their time coefficient *x* varied with bubble diameter. They concluded that as the bubble diameter increases (i.e., the enlarged ratio of bubble size to microelectrode size), the *x* value gradually decreases, which means that the bubble growth rate decreases (Fig. 5b). This demonstrates the necessity of designing electrodes that can detach bubbles when they are still in micro size.

**Fig. 6:**
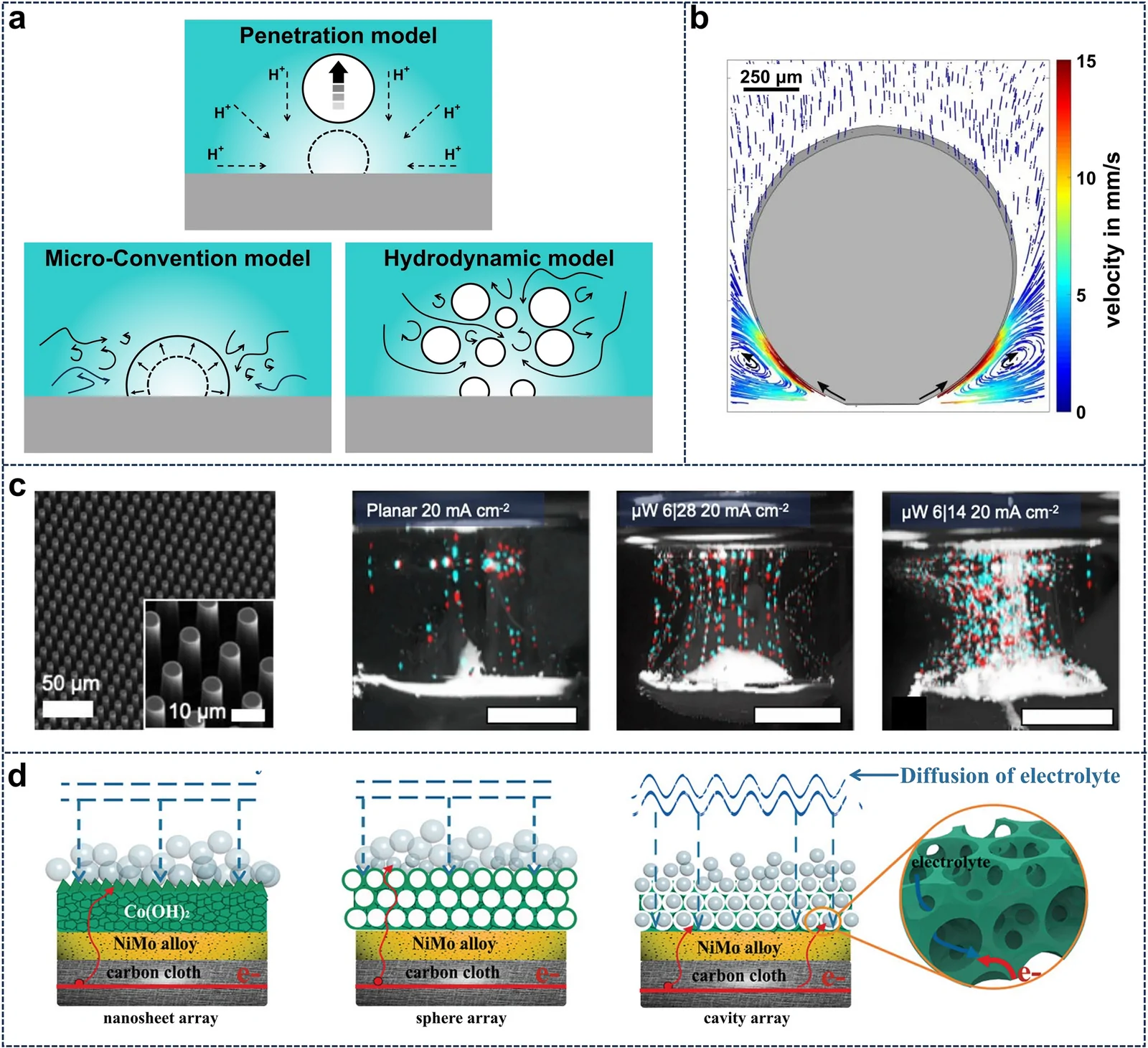
**a** Schematic diagram of three mass transfer models on GER electrodes: penetration model, micro-convention model and hydrodynamic model, respectively. **b** Particle trajectories and corresponding velocities around the growing bubble [42]. Reproduced with permission. Copyright 2018, Royal Society of Chemistry. **c** SEM image of the micro-sized arrays electrode and Bubble release images of three specifications of electrodes during OER process [44]. Reproduced with permission. Copyright 2021, Royal Society of Chemistry. **d** Diagram of mass transfer processes for different surface structures [45]. Reproduced with permission. Copyright 2021, Wiley–VCH

To elucidate the effect of electrode geometry on the dynamics of bubble evolution, Qin et al. [38], respectively, constructed Pt electrodes with nanoarray, smooth and nanoporous surfaces (Fig. 5c, above, nanoarray; lower left, smooth; lower right, nanoporous). The results show that the bubbles generated on the nanoarray electrodes have the smallest bubble detach size and the shortest growth time (Fig. 6d, left). In addition, compared with the other two electrodes, the nanoarray electrode exhibits the best bubble growth kinetics with the largest time coefficient x of 0.54 (Fig. 6d, right). This finding reinforces the advantages of micro/nanostructured electrodes for GERs.

### Promoting Mass Transfer

Superaerophobic electrodes also significantly promote the transfer of ions and electrons in GER, thereby reducing the energy loss caused by interface barriers and reduced ion concentration in the medium during the reaction process. In GERs, mass transfer is mainly the transfer of ions from the electrolyte to the electrode surface and the detachment of the generated gas from the electrode. There are currently three models for ion transport in GERs (Fig. 6a). The first is the penetration mode proposed by Ibl [39]. In this model, when the bubble detaches from the electrode, the resulting local concentration gradient helps ions to diffuse from the electrolyte to the electrode surface. The second model is micro-convention model developed by Stephan and his co-worker Vogt [40]. The model aims to reveal that as the bubble grows, it pushes away the nearby electrolyte, creating micro-convection around it, thereby enhancing mass transfer. The third model is hydrodynamic model proposed by Janssen [41]. This model can be used to describe the free convection of electrolyte caused by the bubble group detaching from the electrode and rising. This model has advantages in describing the generation of a large number of tiny bubbles in superaerophobic electrodes. In this regard, superaerophobic electrodes with micro/nanostructures can effectively promote mass transfer. Eckert et al. [42] used time-resolved particle tracking velocimetry to provide a new perspective for understanding local mass transfer near bubbles (Fig. 6b). They found that the nonuniform hydrogen concentration and ohmic heating of the electrolyte caused by bubble adhesion to the electrode lead to a surface tension gradient at the bubble interface, thereby inducing convection in the electrolyte. Furthermore, the convection intensity near the bottom electrode of the bubble is much higher than that at the top, inducing larger local micro-vortices.

Based on the above models, it is evident that rapid bubble detachment based on micro/nano electrodes can effectively promote mass transfer. For example, Kempler et al. [43, 44] constructed electrodes with micro-structured arrays to verify the micro-convection effect enhanced by micro array electrodes as shown in Fig. 6b. As shown in Fig. 6c, at the same current density, the μW 6|28 and μW 6|14 samples generated much smaller bubbles than the planar sample. In addition, from the colored dots in Fig. 6c, it can be found that the micro-convection of sample μW 6|14 with smaller microstructures is even more obvious. Zhang et al. [45] constructed a superaerophobic electrode with cavity arrays. Compared with the electrodes with nanosheet arrays (Fig. 6d, left) and sphere arrays (Fig. 6d, middle), the cavity array Co(OH)_2_ surface (Fig. 6d, right) ensures the electrolyte diffuses throughout the electrode and provides a large catalytic surface area. In addition, electrons from the substrate can more easily reach the catalyst-electrolyte interface. These excellent performances in mass transfer together increase the catalytic activity of the electrode.

### Enhancing Catalyst Stability

**Fig. 7:**
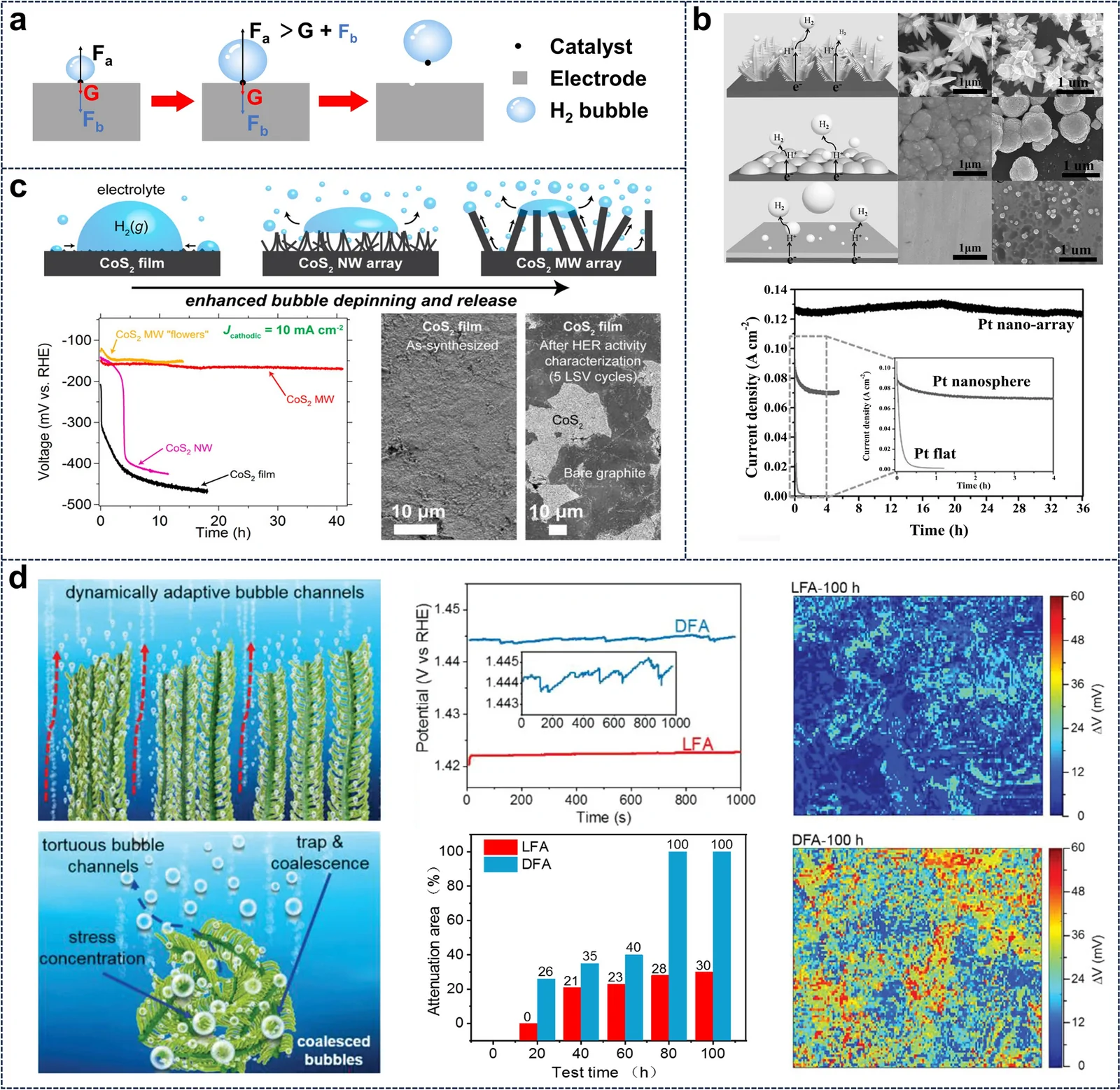
**a** Schematic diagram of the bubble damage to the non-aerophobic electrode when it is generated and released from the electrode (*F*_a_ is adhesion force of bubbles to catalyst, *F*_b_ is the binding force between the catalyst and the electrode, *G* is the gravity of the catalyst). **b** Schematic diagrams of three types of surface topography electrodes (nanoarrays, nanospheres and planar films), SEM images before and after stability tests, and stability test curves of the electrodes [46]. Reproduced with permission. Copyright 2015, Wiley–VCH. **c** Aerophobicity schematic diagrams of CoS_2_ catalysts with different surface structures, the corresponding stability test curves and the SEM images before and after stability tests for CoS_2_ film [47]. Reproduced with permission. Copyright 2015, American Chemical Society. **d** Schematic diagrams of lamellar fern-like alloy aerogel (LFA) and disordered fern-like aerogels (DFA) electrode, the curve of the potential with a current density of 2 mA cm^−2^ over time, the activity attenuation graphs corresponding to different times in the stability test for 1000 mA cm^−2^. The oxygen evolution starting potential mapping images of LFA and DFA electrodes during long-term stability test for 100 h at 1000 mA cm^−2^ current density [48]. Reproduced with permission. Copyright 2024, Wiley–VCH

Superaerophobic electrodes can also significantly enhance the stability of catalysts. As illustrate in Fig. 7a, during the gradual growth of a bubble, the catalyst on the electrode surface is subjected to three forces, namely, the own gravity G, the adhesion force *F*_*a*_ between the catalyst and the bubble, and the binding force *F*_*b*_ between the catalyst and the electrode. These three forces always maintain a dynamic balance when the bubble is small, which can be expressed by an Equation: *F*_*a*_ = *G* + *F*_*b*_. As the bubble grows, *F*_*a*_ also increases, and when the bubble grows to a certain critical size, *F*_*b*_ reaches its maximum, at which point *F*_*a*_ = *G* + *F*_*b*max_. If the bubble continues to grow and eventually detaches from the electrode, the catalyst will break away from the electrode and detach with the bubble together, which will cause the electrode surface to be completely damaged. In contrast, when using superaerophobic electrodes, the bubble has already desorbed from the electrode surface at a relatively small stage, instead of damaging the catalyst on the electrode surface.

To verify the above concepts, Li from Jiang’s team [46], respectively, prepared Pt nanoarray, Pt nanosphere, and Pt flat film on Ti substrates (Fig. 7b). Comparing the SEM images before and after the stability test, the petals of the Pt nanoarray have changed a little, the particles on the surface of the nanospheres have decreased, the overall damage to both is not significant. However, the Pt flat has been damaged greatly. The Pt film has shrunk to a few isolated nanoparticles, and even some nanoparticles have been lost and left some holes on the substrates. It can also be seen from the current density of the stability test that the current densities of both Pt nanosphere and Pt flat samples have been greatly reduced, while the nanoarray has remained relatively stable. Faber et al. [47] prepared CoS_2_ films, nanowire arrays, and microwire arrays with progressively increasing aerophobicity and tested their stability. They found that the CoS_2_ microwire array with the best aerophobicity had the best stability, while the CoS_2_ film mostly detached after only 5 LSV cycles (Fig. 7c). Wang et al. [48] prepared lamellar fern-like alloy aerogel (LFA) and disordered fern-like aerogels (DFA) electrode and found that the DFA electrode was extremely unstable at the initial potential of the stability test. In addition, the activity attenuation after the stability test was significantly greater than that of the LFA, which can be demonstrated by the warm-toned area of the oxygen evolution starting potential mapping image (Fig. 7d).

## Bubble Dynamics and Construction of Superaerophobic Electrodes

Based on the above viewpoints, it is very necessary to use superaerophobic electrodes for GERs. In this section, the theoretical basis of superaerophobicity will be introduced, and the construction method of superaerophobic electrodes will be explored.

### Interfacial Wettability Theory

**Fig. 8:**
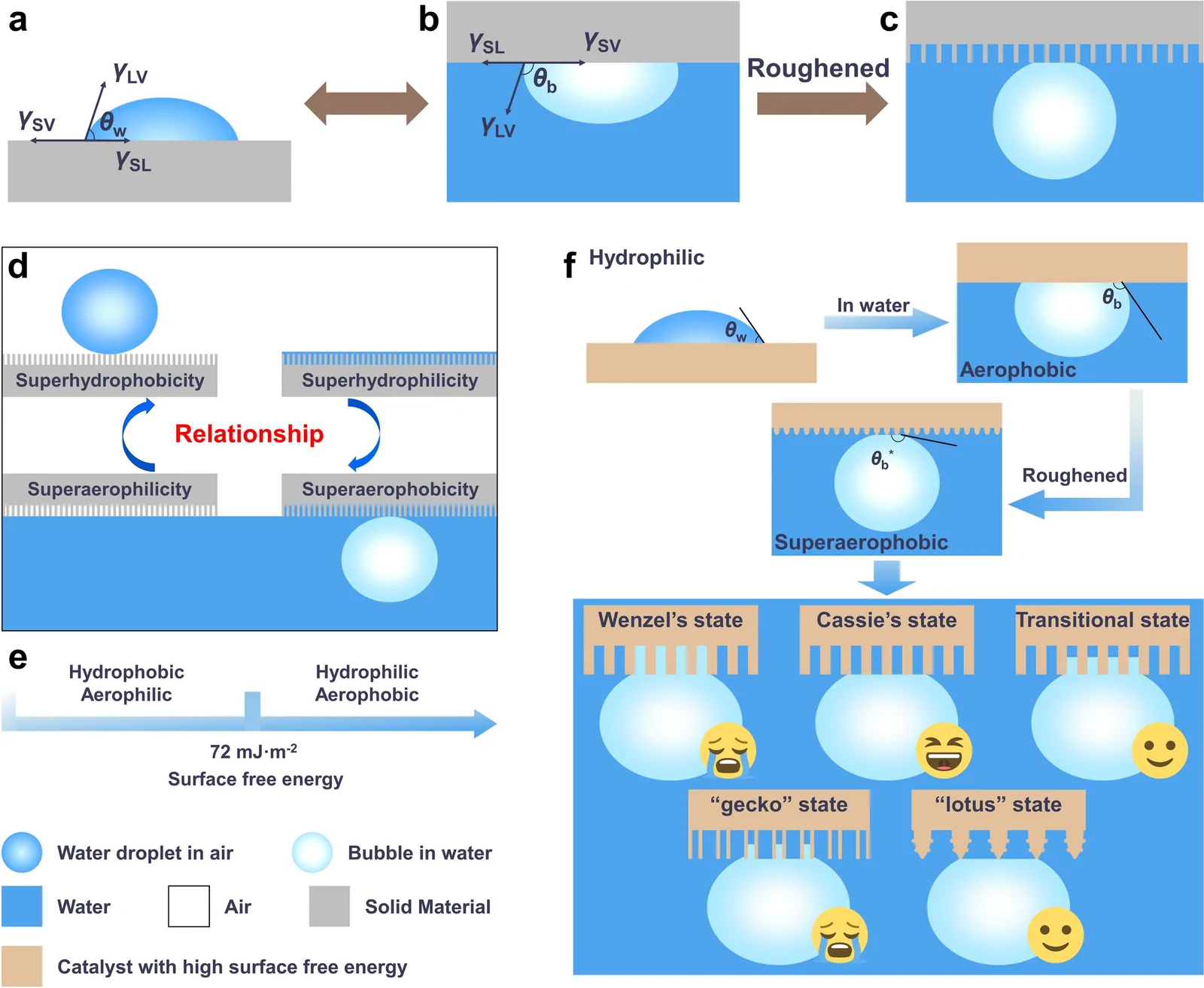
**a** Wetting behavior of a droplet on a solid substrate (Young’s mode) and **b** its corresponding contact mode with bubble under water. **c** Achieving superaerophobicity through roughing on the aerophobic surface. **d** The relationship between superhydrophobicity and superaerophilicity, superhydrophilicity and superaerophobicity: a superhydrophobic surface is superaerophilicity in water, and a superhydrophilic surface is superaerophobicity in water. **e** Wetting behavior based on surface free energy and water surface tension. **f** Strategy of constructing superaerophobic electrode

When water comes into contact with a solid in air, the water molecules that have just come into contact with the solid surface must compete with the gas already present on the solid surface [49]. The intermolecular forces between the solid, liquid, and gas phases will determine the final behavior of the water droplet, which is specifically expressed as the contact angle (CA) [50]. CA is the angle at which the tensions of the three phases at the interface reach equilibrium. The contact angle and the interfacial tension between the three phases satisfy Young’s Eq. (Eq. 11) [13]:

11 $$cos {\theta }_{w} = \frac{{\gamma }_{SV }- {\gamma }_{SL}}{{\gamma }_{LV}}$$

where *θ*_w_ is the water contact angle, *γ*_SV_ is the solid–gas interface energy, *γ*_SL_ is the solid–liquid interface free energy, and *γ*_LV_ is the liquid–gas interface energy (Fig. 8a). The wettability of bubbles on the solid interface in water can be approximately considered to be complementary to the wetting of water on the solid surface in air [51]. The bubble contact angle can also be obtained by Young’s Eq. (Eq. 12):

12 $$cos {\theta }_{b} = \frac{{\gamma }_{SL }- {\gamma }_{SV}}{{\gamma }_{LV}}$$

Further, it can be concluded that (Eq. 13):

13 $${\theta }_{b} = 180^\circ - {\theta }_{w}$$

The above is the relationship between the water contact angle and the bubble contact angle under ideal conditions. Therefore, we can consider the hydrophilic interface to be aerophobic, while the hydrophobic interface to be aerophilic (Fig. 8b).

For rough surfaces, the Wenzel (Eq. 14) [15] and the Cassie-Baxter Equation (Eq. 15) [16] can be introduced to calculate the relationship of the CA between the rough surface and the apparent surface:

14 $$cos {\alpha }^{*} = r cos \alpha$$

15 $$cos {\alpha }^{*} = -1 + {f}_{s} (cos \alpha + 1)$$

where α is the apparent CA of the solid surface, α^*^ is the apparent CA on the roughened solid surface, r is the surface roughness factor, and *f*_s_ is the solid fraction of the contact area. For aerophilic surfaces, the wettability of solid in water follows the Wenzel state (Eq. 14). Increasing the roughness leads to a larger α^*^ and promote the transition from aerophilicity to superaerophilicity. Conversely, increasing the roughness results in superaerophobicity for aerophobic surfaces (Cassie-Baxter state, Eq. 15, Fig. 8c). In this case, bubbles can easily detach from the surface due to the discontinuity of the three-phase contact line (TPCL), minimizing bubble adhesion.

In summary, hydrophobic surfaces exhibit hydrophilicity underwater, while hydrophilic surfaces exhibit hydrophobicity underwater (Fig. 8d). The hydrophilicity/hydrophobicity of solid materials depends on the relative strength of solid–gas interface interactions and solid–liquid interface interactions. Generally, materials with higher surface free energy tend to exhibit hydrophilicity and hydrophobicity underwater; while materials with lower surface free energy tend to exhibit hydrophobicity and hydrophilicity. The surface tension of water (~ 72 mN m^−1^ [52]) is often used as a reference for assessing wettability trends. When the surface free energy of a material exceeds 72 mJ m^−2^, the material is hydrophilic/hydrophobic; conversely, when the surface free energy is below 72 mJ m^−2^, the material exhibits hydrophobic/hydrophilicity (Fig. 8e) [26].

### Construction of Superaerophobic Electrode

Based on the theoretical summary above, a general strategy for constructing superaerophobic electrodes can be derived (Fig. 8f). Typically, roughening is applied to the hydrophilic/aerophobic catalyst which has a high surface free energy. By increasing the surface roughness, electrodes can effectively transition from aerophobic to superaerophobic states [53, 54]. Jiang’s team has summarized five typical wetting states: Wenzel’s state, Cassis’s state, transitional state, “Gecko” state, and “lotus” state [55–57].

In Wenzel’s state, the bubble is pinned on the surface of the rough structure catalyst in contact, but because of the mutual adsorption between the bubble and the gas in the microstructure, it cannot slide freely on the surface, although it may show a high CA. Thus, this state is not suitable for constructing a superaerophobic electrode.

In Cassie’s state, the bubble adopts a non-wetting contact mode on the catalyst surface, and the discontinuity of the solid–liquid-gas TPCL makes it easy for the bubbles to fall off the surface. Therefore, this state is an ideal state for constructing a superaerophobic electrode.

As a transitional state between Wenzel’s and Cassie’s states, its suitability as a superaerophobic catalyst must be determined through actual test performance.

Another state with superaerophobicity is “Gecko” state. Unlike the Cassie’s state, where water is trapped in the rough catalyst and linked to the electrolyte (open state), in the gecko state, in addition to the open water mentioned above, there is also water that is completely sealed in the microstructure. The three-phase linear discontinuity formed by the two trapped waters makes the electrode superaerophobic, while the negative pressure generated by the microstructure sealing water makes it difficult for bubbles to detach from the electrode surface, so the “gecko” state is not suitable for superaerophobic electrodes.

As a unique case of Cassie’s state, the “lotus” state exhibits superaerophobic due to its micro/nano hierarchical structure, thereby minimizing the solid–liquid-gas TPCL and reducing the bubble adhesion force. However, a single micro- or nano-scale surface structures do not inherently guarantee bubble repellence. If the surface of a solid material is not sufficiently hydrophilic, or if partial wetting and gas trapping occur inside a rough structure, capillary pinning and extended three-phase contact lines may actually promote bubble adhesion. So extra attention should be paid to regulating both surface chemistry and structural hierarchy in electrode design.

While various micro/nanostructured construction strategies have been developed, an essential scientific question concerns how structural parameters quantitatively influence bubble detachment behavior and electrode performance. Recent research is working to move our understanding of this question from qualitative observation to quantitative modeling. For example, Kou et al. [58] constructed nickel electrodes with periodically ordered porous structures using 3D printing technology. They found that periodically porous three-dimensional electrodes can effectively reduce bubble accumulation and detachment radius by optimizing pore size and gas channels to regulate bubble movement. Ren et al. [59] demonstrated through simulation that, by controlling the size of nanocones compared to planes, the nanocone surface not only reduces the contact area between bubbles and solids but also changes the direction of adhesion force (no longer completely opposite to buoyancy), thus significantly shortening the bubble adhesion time. More importantly, by controlling the size of the nanocones, they achieved control over the bubble detachment diameter, confirming the direct correlation between nanoscale size and bubble detachment behavior. Besides, the most direct quantitative relationship comes from the work of Hu et al. [60]. They modified the surface gas-repellency by precisely controlling the dendrite tip diameter (1–35 μm) of porous copper. Their study found a positive correlation between the dendrite tip diameter and bubble adhesion force and bubble detachment radius. When the dendrite tip diameter was at its minimum, the bubble adhesion force approached zero, and the detachment radius reached its minimum, thus achieving a minimum overpotential of 178 mV in the hydrogen evolution reaction. Collectively, structural dimensions serve as critical design parameters that directly regulate interfacial adhesion and bubble detachment radius. Quantitative correlations between geometry and bubble dynamics mark a transition from empirical morphology control to predictive structural engineering.

### Characterization Techniques of Bubble Dynamics for Superaerophobic Interfaces

After constructing the superhydrophobic surface, characterizing its superhydrophobicity and elucidating its bubble control behavior is also an important step. Wettability measurement is a primary criterion for evaluating superhydrophobicity. Surface free energy calculations provide theoretical support for the formation of superhydrophobic interfaces. Water contact angle, underwater bubble contact angle, sliding angle, and bubble adhesion force analysis are widely used to quantify the interfacial wetting state and distinguish between Wenzel and Cassie states. In addition to static wettability assessment, in situ visualization techniques allow direct observation of bubble evolution [61]. High-speed optical imaging and in-situ microscopy monitor bubble nucleation, growth, aggregation, and desorption. Advanced methods such as surface plasmon resonance microscopy (SPR) [62–64], and total internal reflection imaging enable high-resolution detection of the early stages of interfacial bubble formation. Furthermore, quantitative analysis of bubble coverage and gas release kinetics is crucial for correlating superhydrophobicity with HER performance. Image-based surface coverage analysis, combined with electrochemical diagnostic methods such as impedance spectroscopy, provides in-depth understanding of the mass transfer resistance caused by bubbles. These multimodal characterization methods collectively establish the structure-wettability-bubble kinetics relationship controlling the function of superhydrophobic electrodes.

### Failure Mechanisms of Superaerophobic Electrode

Although advanced in situ characterization techniques have greatly deepened our understanding of the bubble dynamics at superaerophobic interfaces, long-term stability under actual operating conditions has not been fully addressed. During the long process of GER, especially in harsh electrochemical environments such as high current density, strong acids or bases, and high salts, the performance fails due to various reasons.

Firstly, the degradation caused by continuous bubble impact is the main degradation pathway. The nano-structured array is prone to be affected by the mechanical stress generated by rapid bubble nucleation, growth and detachment. Repeated bubble scouring under high current density may cause the bending, collapse or delamination of the nano-structure, resulting in the loss of roughness and subsequently the weakening of surface wettability.

Secondly, in harsh electrolyte environments, the catalyst may be poisoned and surface passivated. Under high salinity or natural seawater conditions, aggressive ions (such as Cl^−^, ${SO}_{4}^{2-}$, Mg^2+^, Ca^2+^) will adsorb on the active sites or participate in side reactions. These processes may form insulating precipitates or corrosive intermediate products on the electrode surface, blocking the catalytic sites, thereby reducing intrinsic activity and bubble release performance [65].

Thirdly, element loss and component degradation will further accelerate the performance decline. Transition metals or heteroatoms in multi-component catalysts may dissolve, re-deposit or undergo valence state changes during the long-term HER process. The loss or redistribution of these key elements will disrupt the original electronic structure and coordination environment, leading to a decrease in active site density and impaired catalytic kinetics.

In summary, the interaction between mechanical damage, chemical corrosion and component instability determines the long-term failure of the electrode. Understanding these degradation pathways is crucial for guiding the design of robust structures and stable interface chemistry for practical hydrogen evolution applications.

## Strategy of Preparing Superaerophobic Electrodes for HER

**Fig. 9:**
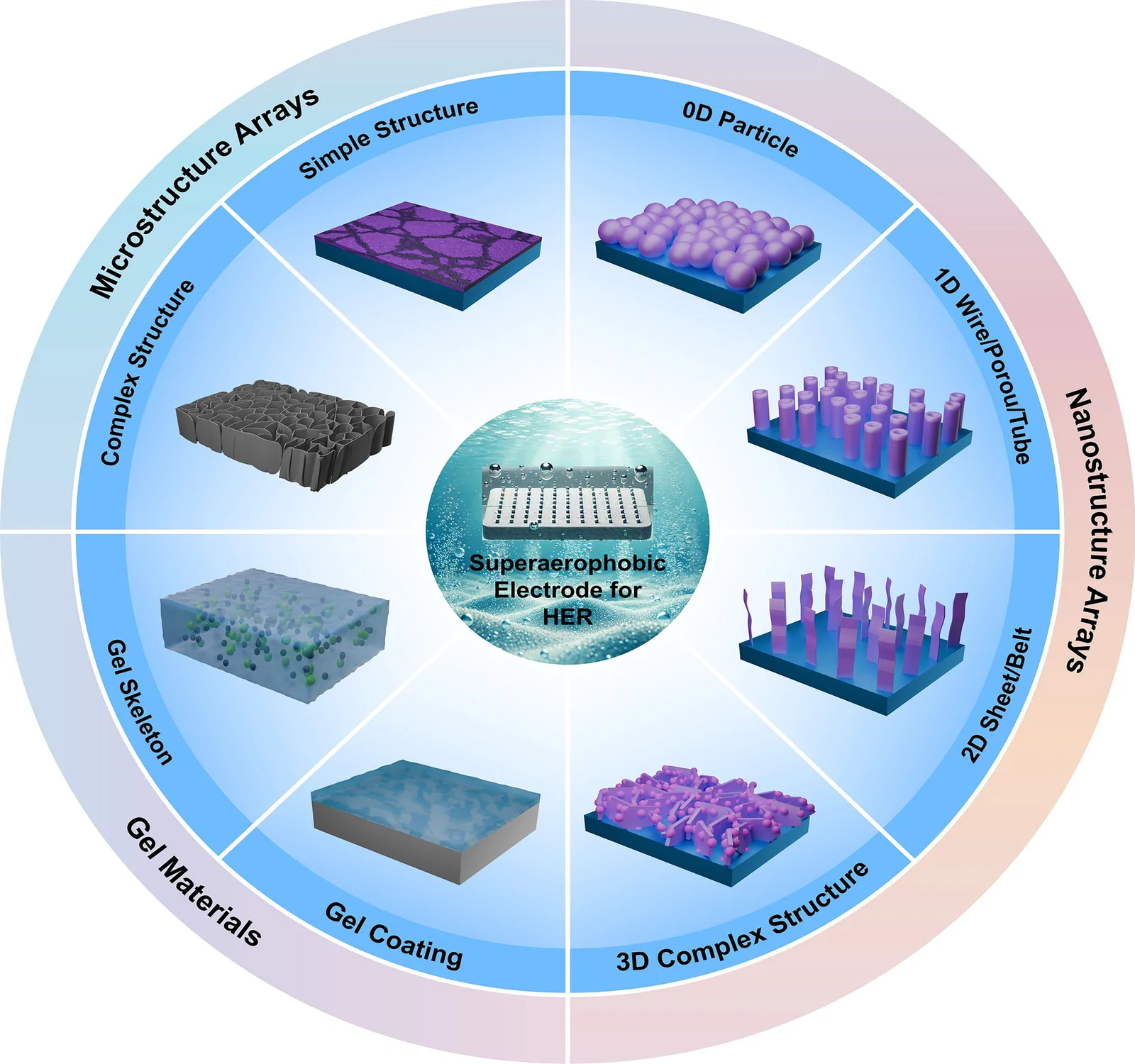
Overview of micro/nanostructure and composition design strategies for superaerophobic electrodes for HER

The above sections discussed the mechanism and methods of constructing micro/nanostructured electrodes to achieve superhydrophobicity. In this section, in addition to reviewing the construction of micro/nanostructures on the electrode surface to achieve superaerophobicity, the spreading of hydrophilic gels on superaerophobic electrodes will also be introduced (Fig. 9).

### Microstructure Arrays

**Fig. 10:**
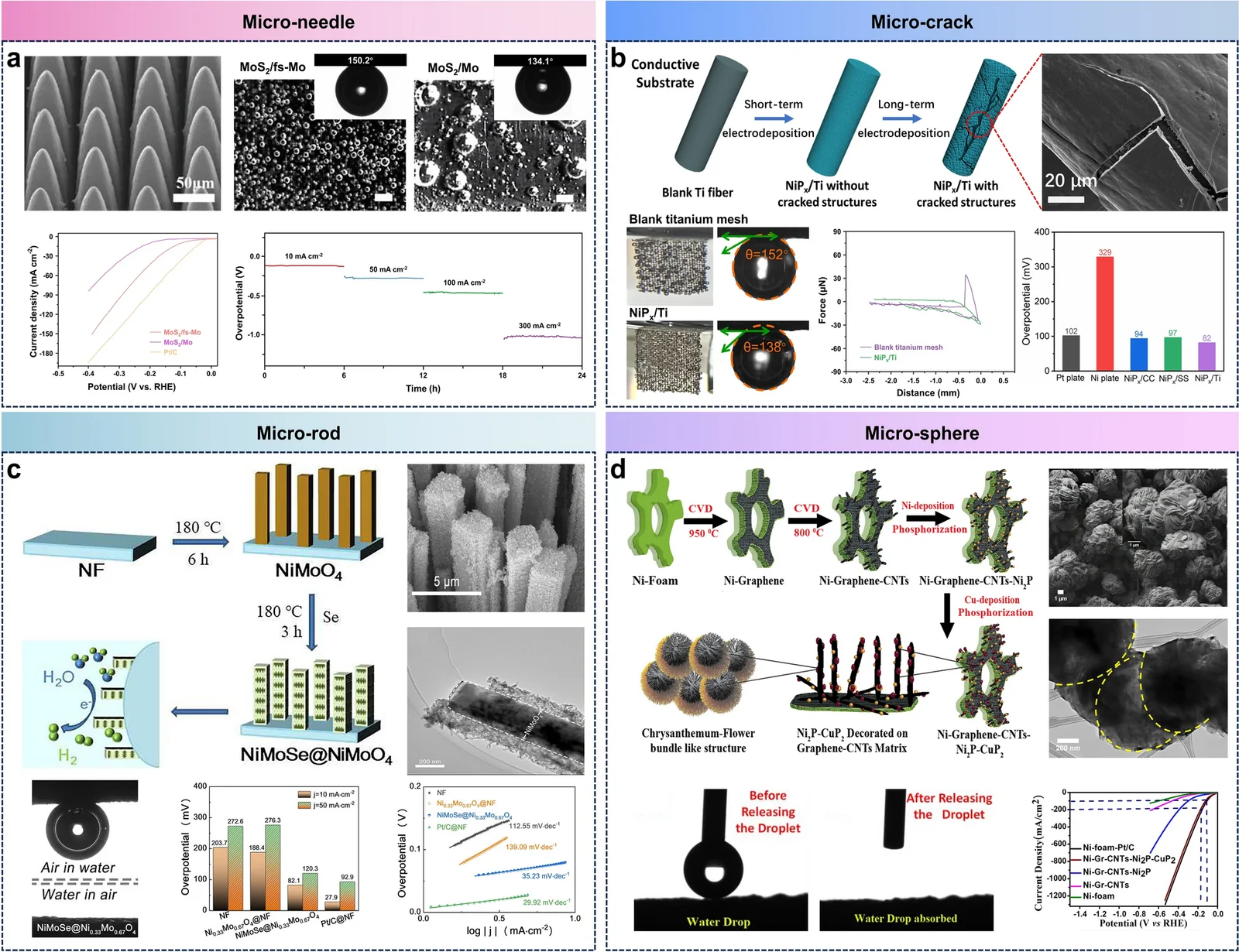
Strategies of preparing superaerophobic simple micro-structured array electrodes for HER. **a** Surface morphology, superaerophobicity, HER property, and stability of micro-needle-structured array electrode [67]. Reproduced with permission. Copyright 2024, Elsevier. **b** Preparation schematic, surface morphology, superaerophobicity, and HER property of micro-crack-structured array electrode [68]. Reproduced with permission. Copyright 2024, Wiley–VCH. **c** Preparation schematic, surface morphology, superhydrophilicity/superaerophobicity, and HER property of micro-rod-structured array electrode [69]. Reproduced with permission. Copyright 2022, Elsevier. **d** Preparation schematic, surface morphologies, superhydrophilicity, and HER property of micro-sphere-structured array electrode [72]. Reproduced with permission. Copyright 2021, American Chemical Society

As an effective strategy to achieve superaerophobicity, constructing electrodes with microstructure arrays becomes the simplest solution. According to the complexity of preparation and microstructure array, it can be divided into simple microstructure and complex microstructure. Tabel 1 summarizes the catalytic performance of various superaerophobic electrodes with microstructures. Simple structures include laser-etched micro-needle arrays, crack structures, and other flake-, spherical-, and rod-like structures that spontaneously crystallize during their synthesis. These structures are relatively easy to prepare, making them an ideal way for scientists to explore the superaerophobic electrodes in the early stages. For example, Yang’s team [66] etched a micro-needle array with a diameter of ~ 40 μm on a Ni substrate with the femtosecond laser. This rough structure of the micro-needle array makes the contact of the bubble in the Cassie’s state, making the TPCL discontinuous, thus showing a superhydrophilic/superaerophobic state. After that, based on the above results, they combined femtosecond laser technology and hydrothermal method to prepare a dual-scale structured MoS_2_ electrode (Fig. 10a) [67]. The nanoscale MoS_2_ catalyst further enhances the superaerophobicity of the electrode (150.2°) and promotes the rapid growth and detachment of bubbles, and mass transfer of the electrolyte.

**Table 1:** Electrochemical properties of superaerophobic electrodes with microstructures

Catalyst	Electrolyte	Electrode	Aerophobic structure	*η*_10_ (mV)	*η* at larger current density (mV@mA cm^−2^)	Stability (h@mA cm^−2^)	References
NiO/Ni	1 M KOH	Ni substrate	Micro-needle	57	387@150	6@10 + 6@30 + 6@50 + 6@100 + 6@300 + 6@500	[66]
MoS_2_	1 M KOH	Mo substrate	Micro-needle	99	383@150	6@10 + 6@50 + 6@100 + 6@300	[67]
NiP_*x*_	1 M KOH	Ti mesh	Micro-crack	82	231@500	150@500	[68]
NiMoSe@NiMoO_4_	1 M KOH	Ni foam	Micro-rod	82	140@100	20@10	[69]
Cu_3_P	1 M KOH	Ni foam	Micro-sheet	130	270@120	20@10	[70]
PNi/Ni	1 M KOH	Chinese rice paper	Micro-sphere	87	138@100	100@10	[71]
Ni-graphene-CNTs-Ni_2_P − CuP_2_	0.5 M H_2_SO_4_	Ni foam	Micro-sphere	12	174@200	80@100 + 80@200 + 80@500	[72]
CoP	0.5 M H_2_SO_4_	Si wafer	Micro-pore	64	386@500	6@10 + 6@30 + 6@50 + 6@100 + 6@300 + 6@500	[73]
Ni-NiP	0.5 M H_2_SO_4_ 1 M KOH 1 M KOH/seawater	Natural camphor pine wood	Micro-pore	– – –	204@1000 269@1000 309@1000	50@500 50@500 –	[74]
NiCoP	1 M KOH	Pinewood-derived carbon	Micro-pore	–	145@1000	1000@1000	[75]
Ni@Cu	0.5 M H_2_SO_4_	Ni foam	Micro-pore	123.5	265.5@100	50@10	[76]
Co–N–C	0.5 M H_2_SO_4_	Aligned porous carbon film	Micro-pore	–	343@1000	32@1000	[77]
Ni-Mo-B	1 M KOH	commercial melamine sponge	Micro-pore	18	257@500	20@5000	[78]
Ni_0.75_Fe_0.25_	1 M KOH	Ni foam	Micro-pore	37	180@250	55@100	[79]
CoP	1 M KOH	Stainless steel mesh	Micro-flower	43	315@400	–	[80]
NiP_2_@MoO_2_/Co(Ni)MoO_4_	1 M KOH	Ni foam	Micro-flower	–	297@1000	650@200	[81]
Ni-Graphene CNTs-Sn_4_P_3_	0.5 M H_2_SO_4_	Ni foam	Micro-flower	62	450@500	5@100 + 5@200 + 5@400	[82]

Sun et al. [68] prepared the electrode for HER by loading phosphorus-doped nickel metal (NiP_*x*_) onto a Ti mesh using electrophoretic deposition. They found that comparing with the electrode deposited for 15 min, the NiP_*x*_ on the surface of the sample deposited for 4 h would spontaneously form cracks (Fig. 10b). These cracks can not only significantly increase the bubble contact angle (increased from 138° to 152°) and reduce the adhesion forces (decrease from 42 μN to virtually 0) on the catalyst surface, making the generated H_2_ bubbles much smaller than those on Pt plate and bare Ti mesh, but also reduce the overpotential.

He et al. [69] synthesized superaerophobic NiMoSe@NiMoO_4_ micro-rods on Ni foam using a hydrothermal method and selenization, which exhibited good superaerophobicity (with the bubble contact angle of 152.3°) and catalytic activity (Fig. 10c).

Riyajuddin from Ghosh’s team [72] electrochemically deposited Ni_2_P and Cu_2_P on Ni foam with graphene and carbon nanotubes (CNTs) deposited by chemical vapor phase (CVD). The in situ formed micro-sphere graphene-CNTs–Ni_2_P–CuP_2_ catalyst on Ni foam endows the electrode with superhydrophilicity and superaerophobicity (Fig. 10d), allowing the bubbles to detach quickly and re-expose the active sites, thus achieving excellent HER performance.

**Fig. 11:**
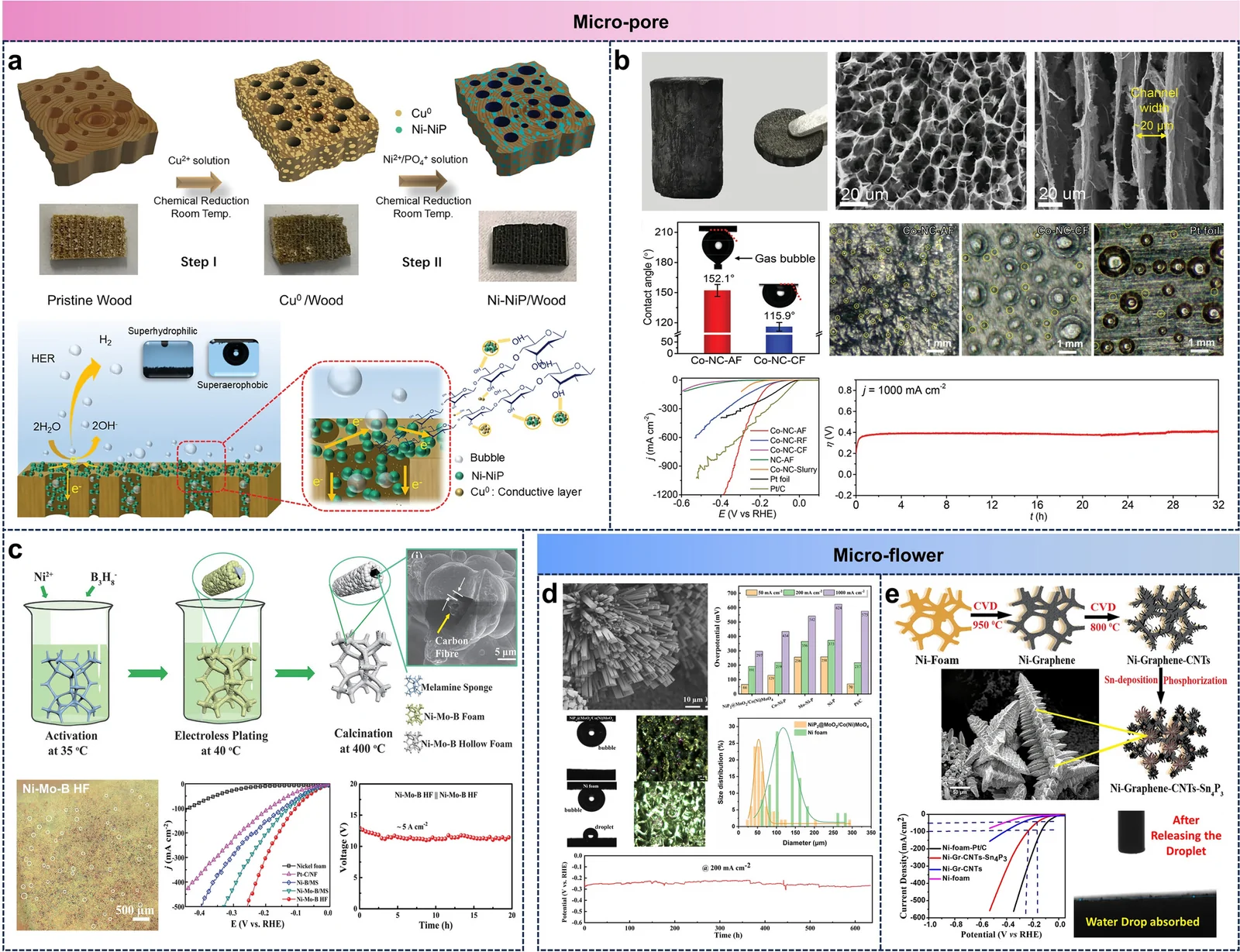
Strategies of preparing superaerophobic complex micro-structured array electrodes for HER. **a** Preparation schematic, superhydrophilicity/superaerophobicity of micro-pore-structured array electrode [74]. Reproduced with permission. Copyright 2024, Wiley–VCH. **b** Optical image, surface morphologies, superhydrophilicity/superaerophobicity, bubble size during HER, HER property, and stability of micro-pore-structured array electrode [77]. Reproduced with permission. Copyright 2021, Wiley–VCH. **c** Preparation schematic, surface morphology, bubble size during HER, HER property, and stability of micro-pore-structured array electrode [78]. Reproduced with permission. Copyright 2022, Wiley–VCH. **d** Surface morphology, HER property, superhydrophilicity/superaerophobicity, bubble size and its distribution during HER, and stability of micro-flower structured array electrode [81]. Reproduced with permission. Copyright 2022, Elsevier. **e** Preparation schematic, surface morphology, HER property and superhydrophilicity of micro-flower structured array electrode [82]. Reproduced with permission. Copyright 2022, American Chemical Society

Complex structures are usually porous structures with three-dimensional microstructures. Such structures are difficult to prepare but have strong superaerophobic properties. Ling et al. [74] innovatively used natural camphor pine wood with rich porous structure and abundant hydroxyl groups as the base electrode material, deposited copper directly on the wood surface, and then loaded Ni-NiP catalyst at room temperature. This method circumvents the defect that carbonized wood will cause the loss of hydroxyl groups. The resulting electrode inherits the superhydrophilic and superaerophobic properties of the original wood, effectively promoting mass and charge transfer. It demonstrates high activity and excellent stability in acidic, alkali, and seawater conditions (Fig. 11a).

Liu [77] designed an aligned porous carbon film embedded with Co–N–C single-atom catalysts through vertical freezing method, as shown in Fig. 11b. The porous aerogel electrode not only increases the surface area, allowing it to load more Co–N–C single-atom catalysts; it also makes it superaerophobic (with the bubble contact angle of 152.1°), promoting electrolyte wetting and ensuring the timely removal of the evolving H_2_ gas bubbles; in addition, the multiscale porosity of the carbon frameworks with vertically aligned micro-channels provide promoted mass transfer under high productivity and ultrathick electrode conditions.

Liu et al. [78] loaded the Ni–Mo–B catalyst onto commercial melamine sponge and then decomposed it at high temperature to produce Ni–Mo–B hollow foam (Ni–Mo–B HF) electrode (Fig. 11c). Thanks to the porous structure obtained after the decomposition of melamine sponge, it can buffer the stress/strain or volume expansion caused by bubble desorption and ensure the cyclic stability during the catalytic process. The porous and particle structure makes Ni–Mo–B HF electrode exhibits excellent superaerophobicity, making H_2_ bubbles it produces at a current density of 100 mA cm^−2^ much smaller than those of Ni foam (The diameter of the H_2_ bubbles generated by Ni–Mo–B HF electrode is 50–80 μm, while the diameter generated by Ni foam is 500 μm). In addition, the micro-channels in the foam can also provide additional ventilation paths for gaseous products, further promoting the mass transfer of the electrode during water electrolysis. These microstructure-derived properties together give the Ni–Mo–B HF electrode excellent catalytic performance: it has an overpotential far lower than that of commercial Pt/C electrodes, and can still work for 20 h at an ultra-high current density of 5 A cm^−2^.

Xu et al. [81] synthesized NiP_2_@MoO_2_/Co(Ni)MoO_4_ micro-flowers on Ni foam, which exhibits excellent catalytic activity and stability. This is attributed to the superaerophobicity of the micro-structure (with the bubble contact angle of 149.5 ± 2°), which significantly reduces the size of the generated bubbles (decrease from 65 to 125 μm) (Fig. 11d).

In addition to preparing micro-sphere graphene-CNTs–Ni_2_P–CuP_2_ catalyst for overall water splitting, Riyajuddin et al. [82] further synthesized graphene-CNTs–Sn_3_P_4_ catalyst on the same Ni-graphene-CNTs matrix by hydrothermal method. This catalyst exhibits micro-flower structures, which makes the electrode superhydrophilic. Water droplets can be quickly absorbed into the electrode, which is conducive to the rapid desorption of bubbles and the replenishment of electrolytes (Fig. 11e).

Microstructure arrays can provide directional bubble transport paths, and their larger size also provides strong mechanical strength; however, their large size limits the improvement of the electrochemically active surface area. Furthermore, at high current densities, microcavities can also become bubble trapping points, leading to localized gas accumulation and partial blockage. Future optimization should focus on hierarchical integration with nanoscale catalytic layers to compensate for the lack of active sites. Precise control of the nanostructure size can further reduce bubble aggregation.

### Nanostructure Arrays

Comparing to microstructure electrodes, the unique porosity, sparsity, and surface activity of nanostructure arrays of electrodes significantly enhance gas transport and reaction performance. During catalysis, nanostructure arrays are able to enhance charge transfer and electrolyte replenishment by optimizing mass transfer pathways. In addition, superaerophobicity achieved by the complex nanostructure arrays can effectively prevent gas bubble attachment and enable rapid detachment and reuse, thus enhancing catalytic efficiency and reaction stability. These advantages make nanostructure arrays ideal for the development of highly efficient and durable electrodes. Table 2 summarized the superaerophobic electrodes with nanostructure arrays and their performance parameters for hydrogen evolution.

**Table 2:** Electrochemical properties of superaerophobic electrodes with nanostructures

Catalyst	Electrolyte	Electrode	Aerophobic structure	*η*_10_ (mV)	*η* at larger current density (mV@mA cm^−2^)	Stability (h@mA cm^−2^)	References
NiMo	6 M KOH	Cu foam	Nanoparticle	–	113@200	100@372	[83]
NiFe	1 M KOH	Carbon cloth	Nanoparticle	19	290@200	150@100	[84]
Co_9_S_8_–Ni_3_S_2_–CNTs	1 M KOH	Ni foam	Nanoparticle	243	429@50	10@10	[85]
Ni–Co–S–P	0.5 M H_2_SO_4_	Carbon fiber cloth	Nanoparticle	78	163@100	25@100	[86]
Pt@Co	1 M KOH	Carbon nanofiber	Nanoparticle	15	145@200	100@100	[87]
Ni_3_S_2_	1 M KOH	Ni foam	Nanoparticle	189	310@100	6000 LSV cycles	[88]
NC@NiNPs	1 M KOH	Glassy carbon electrode	Nanoparticle	74	–	260@1400	[89]
NiCoP/NiMoO_4_	1 M KOH	Ni foam	Nanorod/wire	57	242@350	100@10	[90]
Se–CoS_2_ NW	1 M KOH/0.5 M urea	Carbon fiber	Nanorod/wire	188	514@200	48@10	[91]
NiCoFeP@NiCoP	1 M KOH 0.5 M H_2_SO_4_ 1 M PBS Seawater	Ni foam	Nanorod/wire	77 136 184 400	293@200 410@400 – 693@100	10@10 – – 25@10	[92]
Co_4_N–CeO_2_	1 M KOH	Ni foam	Nanorod/wire	52	151@300	100@24	[93]
Ni Pt–Ni Cr–Ni	1 M KOH	Ti foil	Nanorod/wire	135 57 81	271@100 217@100 243@100	10@18 + 50@18 – –	[35]
NiMoO_4_@NiFeP	1 M KOH 1 M KOH/seawater	Ni foam	Nanorod/wire	58 –	353@500 370@500	– 100@100	[94]
Ni_3_S_2_/VS_2_	1 M KOH	Ni foam	Nanorod/wire	164	350@100	10@50	[95]
Ni_3_S_2_/CNTs	1 M KOH	Ni foam	Nanorod/wire	64	324@300	100@50	[96]
Co_3_(PO_4_)_2_-MoO_3−*x*_/CoMoO_4_	1 M KOH 1 M KOH/seawater Natural seawater	Ni foam	Nanorod/wire	21 24 –	130@500 151@500 –	100@500 100@100 100@500	[97]
Ni_2_P	1 M KOH	Carbon fiber	Nanorod/wire	90	157@50	24@10	[98]
NiMoO_4_–CuO	1 M KOH	Cu foam	Nanorod/wire	–	800@500	24@50	[99]
P–NiMoO_4_	1 M KOH	Ni foam	Nanorod/wire	93	207@70	20@10 + 40@100	[100]
Cu_2_S@NiS@Ni/NiMo	1 M KOH/0.5 M NaCl 1 M KOH/seawater	Cu foam	Nanorod/wire	69 –	190@1000 250@1000	2500@500 2000@500	[101]
Ni_3_N/Ni@W_2_N_3_	1 M KOH	Carbon cloth	Nanorod/wire	66	223@100	200@24	[102]
PBSCF-Ni_3_S_2_	1 M KOH	Ni foam	Nanorod/wire	21	249@1000	500@500	[103]
Ru@NiCo-BH	1 M KOH 1 M PBS 0.5 M H_2_SO_4_	Ni foam	Nanorod/wire	29 68 80	150@150 212@100 224@300	20@100 – –	[104]
Ni_3_S_2_/NiMoS	1 M KOH	Ni foam	Nanorod/wire	–	250@300	14@50	[105]
CoP/CoMoO_4_	1 M KOH	Carbon cloth	Nanorod/wire	86	180@250	5000 LSV cycles	[106]
Ni_3_S_2_@Ni(II)-TC	1 M KOH	Ni foam	Nanorod/wire	166	423@120	24@20	[107]
Co-Ni_*x*_P@C	1 M KOH	Ni-Co foam	Nanorod/wire	55	220@120	24@10	[108]
CoMo/CoTe	1 M KOH 1 M PBS 1 M KOH/0.5 M NaCl 1.0 M PBS/0.5 M NaCl	Carbon cloth	Nanorod/wire	80 107 92 112	600@270 600@118 583@300 600@142	24@10 + 24@50 + 24@100 140@10 135@10 140@10	[109]
FeS	1 M KOH	Ni-Co foam	Nanorod/wire	138	366@400	550@500	[110]
Ni/NiMoN	0.5 M Na_2_SO_4_/0.25 M KH_2_PO_4_/0.25 M K_2_HPO_4_	Cu foam	Nanorod/wire	37	122@50	24@10	[111]
W_18_O_49_	1 M KOH	W wafers	Nanorod/wire	–	–	400@12	[112]
Ag-BHT-MOF	0.5 M H_2_SO_4_	Si/SiO_2_ wafers	Nanorod/wire	105	275@1000	1000 LSV cycles	[113]
CoMoS_*x*_	1 M KOH	Ni foam	Nanopore	89	283@600	100@500	[114]
Poly(EDOT-SuNa)/Ni/Au	1 M KOH	Au substrate	Nanopore	273	265@120	48@10	[115]
Co(OH)_2_/NiMo	1 M KOH	Carbon cloth	Nanopore	32	200@250	24@100	[45]
Ni_2_P–CoOOH	1 M PBS 1 M KOH Seawater	Carbon fiber	Nanotube	20 – 194	887@1500 407@2000 552@1000	100@1200 200@2000 100@100	[116]
FeCoNi-HNTAs	1 M KOH	Ni foam	Nanotube	58	271@150	80@200	[117]
NiCoP–Cr_2_O_3_	1 M NaOH + seawater	Ni foam	Nanosheet	–	257@4000	500@10,000	[118]
Ni_4_Mo/MoO_2_@GF-NVG	1 M KOH	Commercial graphite felt	Nanowire with nanosheets	19	–	100@10	[119]
MoS_2_	0.5 M H_2_SO_4_	Ti foil	Nanosheet	–	500@162	~ 6@170	[120]
Ni–Co–P	1 M KOH 0.5 M H_2_SO_4_	Carbon fiber cloth	Nanosheet	55 74	136@80 174@80	23@100 25@10	[121]
(Ni_0.33_Fe_0.67_)_2_P	1 M KOH	Ni foam	Nanosheet	84	300@200	12@160	[122]
Co(OH)_2_	6 M KOH	Ni foam	Nanosheet	184	553@400	100@100	[123]
NiSe_2_–Ni_5_P_4_	1 M KOH	Ni foam	Nanosheet	65	270@500	100@100	[124]
Ni_2_P	0.5 M H_2_SO_4_	Carbon foil	Nanosheet	63	294@150	11@10	[125]
Mo–Co_9_S_8_	0.5 M H_2_SO_4_ 1 M KOH 0.5 M Na_2_SO_4_	carbon fiber cloth	Nanosheet	98 113 140	290@100 330@100 –	7@24 15@24 –	[126]
Ni–Zn	1 M KOH	Ni foam	Nanosheet	68	147@200	12@100	[127]
Ni_3_N/FeNi_3_N	1 M KOH	Ni foam	Nanosheet	48	117@300	100@50	[128]
Cu–Co–P	1 M KOH, 25 °C 6 M KOH, 80 °C	Ni foam	Nanosheet	65 –	196@500 –	– 220@500	[129]
Co–P	1 M KOH, 25 °C 6 M KOH, 80 °C	Cu foam	Nanosheet	95 –	213@200 –	560@100 240@100	[130]
WS_2_/Ru	1 M KOH	Carbon cloth	Nanosheet	32	154@200	100@10	[131]
NiCoP@Co_0.5_Ni_0.5_Se_2_	1 M KOH	Ni foam	Nanosheet	–	250@671	500@100	[132]
Ru/Co(OH)_2_	1 M KOH	Carbon cloth	Nanosheet	35	75@100	14@500	[133]
Ru–c-CoSe_2_	0.5 M H_2_SO_4_ 1 M KOH	Carbon cloth	Nanosheet	105 97	157@300 301@300	24@10 24@10	[134]
Co_*x*_P, 1 ≦ *x *≦ 2	1 M KOH	Ni foam	Nanosheet	32	468@1000	12@ ~ 850	[34]
NiCoSeP	1 M KOH 0.5 M H_2_SO_4_	Ni foam	Nanosheet	52 49	231@500 130@500	15@500 15@250	[135]
N–CoS_2_	0.5 M H_2_SO_4_	Carbon cloth	Nanosheet	112	153@100	10@100	[136]
FeNi	1 M KOH	Nonwoven stainless-steel fabrics	Nanosheet	110	423@400	18@10	[137]
MoS_2_	0.5 M H_2_SO_4_	Glassy carbon substrate	Nanosheet	293	425@60	–	[138]
WS_2_	0.5 M H_2_SO_4_	p-Si wafers	Nanosheet	200	90@50	20@10	[139]
NiS@NOSC	1 M KOH	Carbon cloth	Nanosheet	64	249@300	6@10 + 6@20 + 6@30 + 6@40	[140]
Co-Ni_3_N	1 M KOH	Ni foam	Nanosheet	–	125@1000	100@800	[141]
NiCoP–NPCNT	1 M KOH	Ni foam	Nanosheet	53	288@300	100@100	[142]
NiFeNb	1 M KOH	Ni foam	Nanosheet	–	487@350	42@12	[143]
Ni_2_P	0.5 M H_2_SO_4_ 1 M KOH 0.5 M PBS	Ni foam	Nanosheet	174 64 77	247@60 208@80 –	65@15 75@10 70@5	[144]
NiCoS_*x*_@CoCH	1 M KOH	Ni foam	Nanosheet	55	438@1000	500@500	[145]
NiFeP@Ni	0.5 M H_2_SO_4_	Carbon nanofiber	Nanosheet	131	267@150	24@20	[146]
Ni_3_FeN@C	1 M KOH 1 M KOH/seawater	Ni foam	Nanosheet	– –	286@800 –	– 500@100	[147]
NiFe LDH@Ni_3_N	1 M KOH	Ni foam	Nanosheet	–	348@1000	100@500	[148]
CoMoS_*x*_	1 M KOH	Ni foam	Nanosheet	84	228@500	100@500	[149]
NiMo/Cu	1 M KOH	Cu foam	Nanosheet	24	198@500	30@30	[150]
P-Ni(OH)_2_/NiMoO_4_	1 M KOH	Ni foam	Nanosheet	60	292@300	30@50	[151]
Pt	0.5 M H_2_SO_4_	Au microgrid	Nanosheet	65	195@153	–	[152]
Ni–Mo	1 M KOH	Ni foam	Nanosheet	35	180@250	1000 LSV cycles	[153]
Ni_2_P/Co(PO_3_)_2_	1 M KOH 1 M KOH/seawater 1 M KOH/wastewater	Ni foam	Nanosheet	25 62 77	217@1000 307@1000 469@500	100@220 60@600 80@225	[154]
Ni_2_P/CoP/P,F	1 M KOH/0.5 M urea	Ni foam	Nanosheet	65	288@300	30@100	[155]
Ru/MXene	1 M KOH	Ni foam	Nanosheet	37	320@1000	14@100	[156]
Pt–MoS_2_	0.5 M H_2_SO_4_	Glassy carbon substrate	Nanosheet	61	149@80	–	[157]
NiCoP	1 M KOH	Ni foam	Nanosheet	79	169@150	10@110	[158]
FeCo@NiCoFeP	1 M KOH	Carbon cloth	Nanosheet	105	343@100	20@20	[159]
Ni–MoO_2_	1 M KOH	Ni foam	Nano wrinkle	49	258@400	60@400	[160]
N–WC	0.5 M H_2_SO_4_	Carbon fiber paper	Nanobelt	89	190@200	10@20 + 10@50	[161]
WS_2_	0.5 M H_2_SO_4_	–	Nanobelt	60	206@100	20@100	[162]
Ni_2_P/MnP_4_	1 M KOH	Carbon fiber	Micro-sheet with nanowires	69	435@5000	180@1000	[163]
RuSA-RuP@NPB	1 M KOH	Carbon nanosphere	Nanoparticle encapsulates nanoparticles	19	74@100	500@1000	[164]
Pt@N–CNC	0.1 M HClO_4_	Glassy carbon substrate	Nanoparticle encapsulates nanoparticles	12	36@50	360@50	[165]
Ni_2_P	1 M KOH	Ni foam	Nanowire with nanosheets	37	744@4000	10@2500	[166]
WS_2_	1 M KOH, 25 °C 1 M KOH, 60 °C	Carbon fiber cloth	Nanowire with nanosheets	– –	360@2000 –	40@500 + 60@1000 + 40@2000 1000@1000	[167]
Mn–MoS_2_	0.5 M H_2_SO_4_	Carbon cloth	Nanowire with nanosheets	130	248@100	10@10 + 100@10 + 200@10	[168]
NiMo	1 M KOH	Ni foam	Nanosheet with nanoparticles	52	191@150	20@10 + 20@100 + 20@200	[169]
Al/P–Co_3_O_4_	1 M KOH	Ni foam	Nanoflower	80	183@80	24@50	[170]
CoS_2_	0.5 M H_2_SO_4_	graphite or glass substrate	Nanoflower	145	221@200	10@10, destroyed	[47]
Ni_2_P–CoP–NiCo_2_O_4_	1 M KOH, 25 °C 6 M KOH, 85 °C	Ni sheet Ni foam	Nanoflower	– –	158@100 –	– 330@2000	[171]
Pt	0.5 M H_2_SO_4_	Ti substrate	Nanoflower	42	84@180	36@120	[46]
Zn-Ni_2_P/Ni_12_P_5_	1 M KOH	Ni foam	Nanoflower	–	367@1000	30@1500	[172]
B-NiMoO_4_	1 M KOH	Ni foam	Nanoflower	23	218@150	60@500	[173]

In this section, the strategies for constructing nanostructure arrays for superhydrophobic electrodes is presented according to the dimension of nanostructures.

#### 0D Nanostructure Arrays

As the primitive structure of superaerophobic electrodes, 0D nanoparticles provide an ideal catalytic interface for HER due to their high specific surface area and abundant active sites. Cassie’s state can construct by precisely tuning the periodic array arrangement of nanoparticles, which enables rapid nucleation and detachment of gas bubbles.

**Fig. 12:**
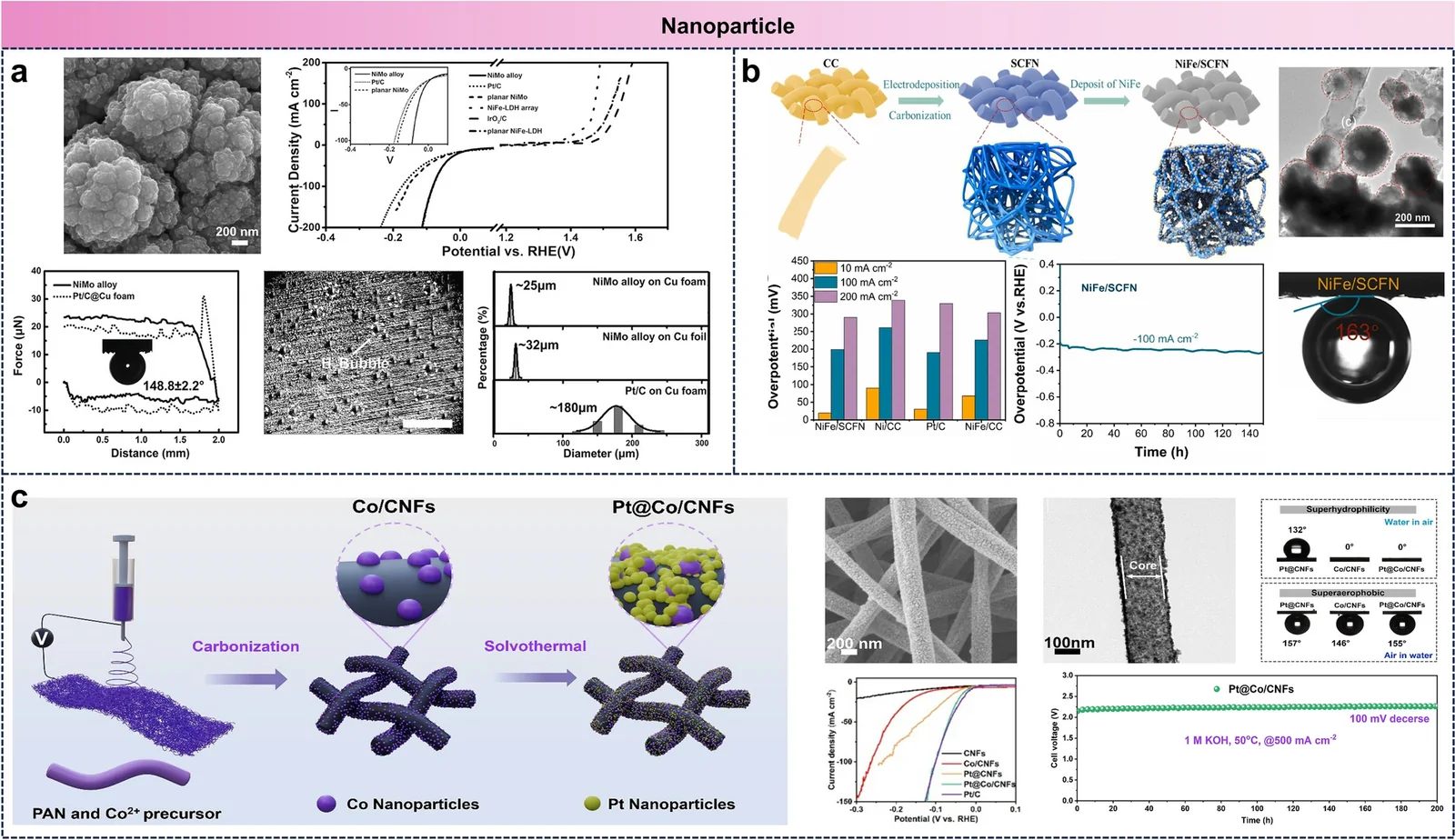
Strategies of preparing superaerophobic 0D nano-structured array electrodes for HER. **a** Surface morphology, HER property, bubble adhesion, bubble size and its distribution during HER of nanoparticle-structured array electrode [83]. Reproduced with permission. Copyright 2016, Wiley–VCH. **b** Preparation schematic, surface morphology, HER property and stability, and superaerophobicity of nanoparticle-structured array electrode [84]. Reproduced with permission. Copyright 2024, Elsevier. **c** Preparation schematic, surface morphology, superhydrophilicity/superaerophobicity, HER property and stability of nanoparticle-structured array electrode [87]. Reproduced with permission. Copyright 2024, Elsevier

For instance, Xu et al. [83] constructed a NiMo alloy superaerophobic electrode with a nanoparticle structure on the cathode surface for hydrogen evolution under alkaline conditions (Fig. 12a). From the polarization curves, it can be found that the nanoparticle NiMo alloy has a much lower overpotential than those of Pt/C film and planar NiMo alloy in HER. Such excellent catalytic performance is attributed not only to the catalytic performance of NiMo alloy itself, but also to the superaerophobicity produced by regulating the nanostructure. Compared with the electrode coated with Pt/C film, the nanoparticle NiMo alloy has lower bubble adhesion, which allows the generated H_2_ bubbles to detach from the electrode surface with an extremely small size of 25 μm, thereby quickly re-exposing the active sites and greatly enhancing the catalytic performance.

Zheng et al. [84] prepared NiFe nanoparticles through electrodeposition on a submicron network carbon cloth. The nanoparticle structure endowed the electrode with excellent gas-repellent properties (with the bubble contact angle of 163°), making its catalytic activity almost equivalent to that of Pt/C catalysts (Fig. 12b).

Guan et al. [87] prepared Co-loaded PAN-based carbon nanofibers as carriers of Pt nanoparticles. The superaerophobicity of the electrode was regulated by changing the size of Pt nanoparticles. The water contact angle and bubble contact angle of the electrode with the best superaerophobicity were 0° and 154°, respectively (Fig. 12c).

#### 1D Nanostructure Arrays

Compared to 0D nanoparticles, 1D nanowire/tube/pore structures exhibit stronger research and application potential. In superhydrophobic electrodes, the unique geometry of these 1D structures can significantly enhance the efficiency of HER. For example, nanowires can resist the destruction of catalysts on the electrode surface due to the rapid generation of a large number of bubbles under high current density because of their good flexibility; nanotube arrays can increase electron mobility by 2–3 orders of magnitude due to their vertical orientation, and their internal hollow structure can be used as a molecular-level electrolyte transport “highway;” nanopores can reduce the retention time of gas bubbles to milliseconds through the synergistic effect of capillary effect and superaerophobicity of the surface, significantly inhibiting the electrode passivation phenomenon.

**Fig. 13:**
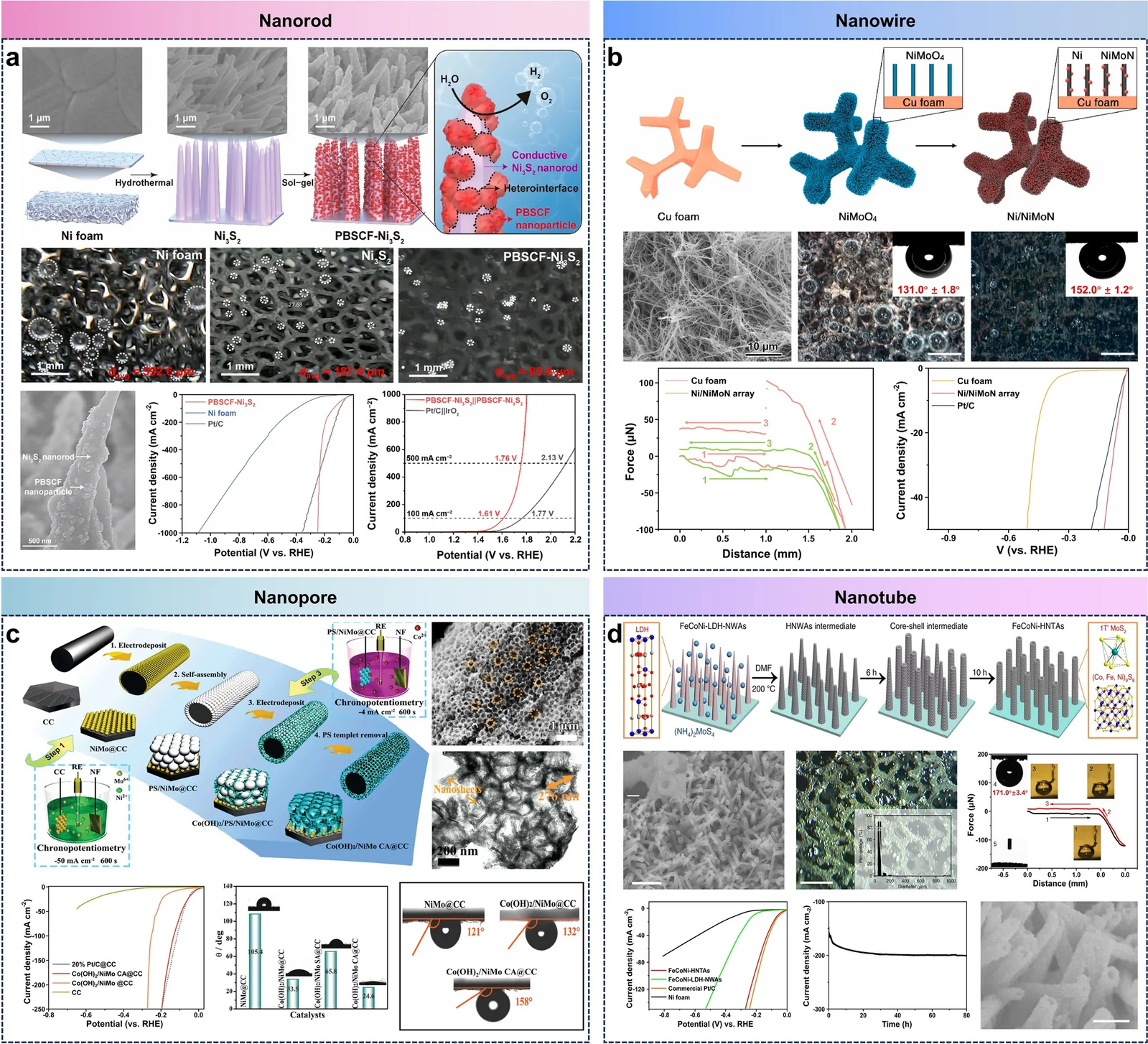
Strategies of preparing superaerophobic 1D nano-structured array electrodes for HER. **a** Preparation schematic, bubble size during HER, surface morphology, HER property and over-all water splitting property of nanorod-structured array electrode [103]. Reproduced with permission. Copyright 2024, American Chemical Society. **b** Preparation schematic, surface morphology, bubble size during HER, bubble adhesion, and HER property of nanowire-structured array electrode [111]. Reproduced with permission. Copyright 2020, Elsevier. **c** Preparation schematic, surface morphologies, HER property and water/bubble contact angles of nanopore-structured array electrode [45]. Reproduced with permission. Copyright 2021, Wiley–VCH. **d** Preparation schematic, surface morphologies, bubble size and its distribution during HER, bubble adhesion, HER property and stability, and surface morphologies after stability test of nanotube-structured array electrode [117]. Reproduced with permission. Copyright 2018, Springer Nature

Jeong et al. [103] used the sol–gel method to load 0D PrBa_0.5_Sr_0.5_Co_1.5_Fe_0.5_O_5+δ_ (PBSCP) nanoparticles onto 1D Ni_3_S_2_ nanorods (Fig. 13a). This 1D nanorod structure endows the electrode excellent superaerophobicity (the average size of the bubbles released from the electrode was 83.6 μm). Besides, the strong electronic coupling effect at the PBSCF/Ni_3_S_2_ heterointerface further enhances the catalytic performance. These two characteristics together greatly enhance the catalytic performance of the PBSCF/Ni_3_S_2_ electrode, making it have an overpotential much lower than that of Pt/C catalyst at high current density.

Shang et al. [111] synthesized NiMoN nanowire arrays decorated with Ni nanoparticles on copper foam as shown in the left of Fig. 13b. The nanowire array imparts the electrode surface with superaerophobic properties (with a bubble contact angle of 152.0° ± 1.2°), thus allowing H_2_ gas bubbles to detach in a small diameter. From the bubble adhesion curve, it can be found that the bubble adhesion of the Ni/NiMoN array with nanowire structure is much smaller than that of the original copper foam. This excellent superaerophobicity makes the catalytic performance even better than that of Pt/C catalysts.

Zhang et al. [45] proposed a strategy to prepare Co(OH)_2_ cavity arrays-encapsulated NiMo alloy on carbon cloth (Fig. 13c). Nanoporous structure enables the electrode to have a bubble contact angle of up to 158°. In addition, the interconnected nano-cavities greatly enhance the diffusion of electrolytes in the electrodes, thereby improving mass transport, as described in Sect. 3.3 and Fig. 7d.

Li and the co-workers [117] designed a hybrid nanotube array catalyst formed by Kirkendall cavitation of ternary Fe, Co, and Ni-based layered double hydroxide nanowire arrays, as shown in Fig. 13d. The electrode with nanotube arrays on the surface exhibits excellent superhydrophobicity: the diameter of the released bubbles is less than 100 μm, the bubble contact angle is as high as 173.1° ± 3.4°, and no adhesion force between the bubbles and the surface. The outstanding superaerophobicity results in excellent hydrogen evolution performance and stability of the catalyst, exhibiting almost identical activity to Pt/C and a surface morphology that remains virtually unchanged after stability testing.

#### 2D Nanostructure Arrays

The 2D nanosheet/belt structures can provide more catalytic active sites through their large surface area, significantly enhancing the reaction activity. The unique structures provide control platforms for the TPCL, which is conducive to the precise control of the superaerophobicity of the electrode. In addition, the 2D sheet/belt structure can ensure that the catalyst is firmly fixed on the substrate surface, which is conducive to the construction of high-performance superaerophobic electrodes under extreme current density.

**Fig. 14:**
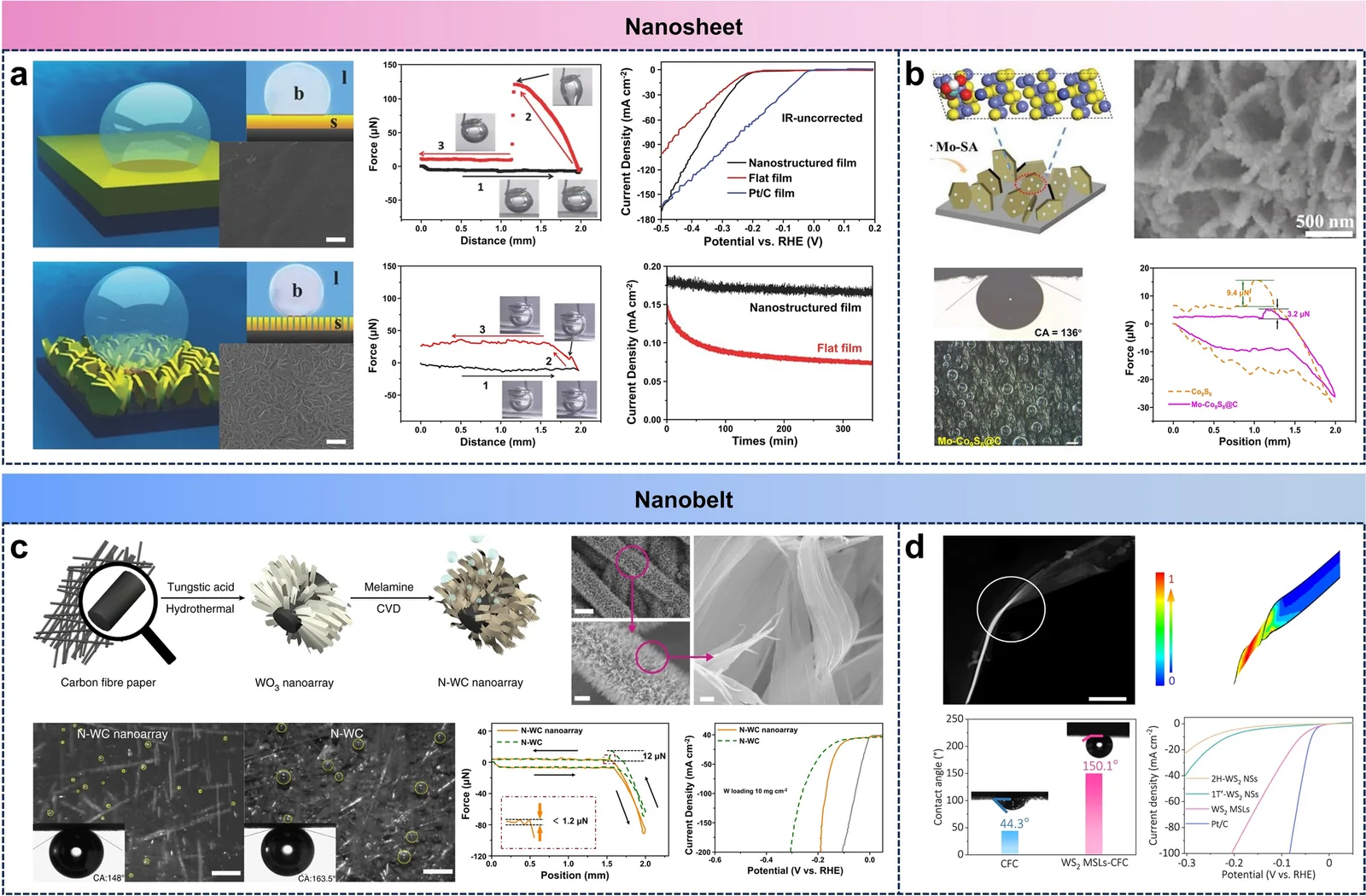
Strategies of preparing superaerophobic 2D nano-structured array electrodes for HER. **a** Schematic illustrations, surface morphologies, bubble adhesions, HER properties and stabilities of nanosheet-structured array electrode compared with flat-structured electrode [120]. Reproduced with permission. Copyright 2014, Wiley–VCH. **b** Schematic illustration, surface morphology, bubble contact angle, bubble size, and bubble adhesion of nanosheet-structured array electrode [126]. Reproduced with permission. Copyright 2020, Wiley–VCH. **c** Preparation schematic, surface morphologies, bubble size, bubble contact angle, and bubble adhesion of nanobelt-structured array electrode [161]. Reproduced with permission. Copyright 2018, Springer Nature. **d** Surface morphologies, finite element calculation of strain, bubble contact angle, and HER property of nanobelt-structured array electrode [162]. Reproduced with permission. Copyright 2021, Springer Nature

As the first team to construct superaerophobic structures for GER, Lu from Jiang’s team [120] prepare nanosheet-structured MoS_2_ on the surface of Ti foil by hydrothermal method (Fig. 14a). The nanosheet structure promotes the discontinuity of TPCL and the superaerophobicity of the electrode, making the diameter of most bubbles smaller than 100 μm when released and the adhesion force of bubbles only 10.8 ± 1.7 μN. It should be noted that that the large surface area of the 2D nanosheets provides a stable and large platform for the attachment of single-atom catalysts. Thus, lots of efforts has been done on loading single-atom catalysts on nanosheet-structured superaerophobic electrodes [174]. For example, Wang and the coworkers [126] synthesized superaerophobic single-atom catalyst by decorating single-atom Mo on Co_9_S_8_ nanosheets for water splitting, as shown in the first and second image of Fig. 14b. The synergistic effect of the single-atom Mo and Co substrate can change the binding energy of the adsorbed intermediate substances and reduce the overpotential of water decomposition. Together with the high bubble contact angle and low bubble adhesion caused by the nanosheet structure, the performance of this catalyst for overall water splitting is superior to that of noble metal Pt/IrO_2_-based catalysts.

In addition to nanosheets, researchers have also developed 2D belt-shaped catalysts with stronger toughness. For instance, Han et al. [161] proposed a nitrogen-doped tungsten carbide electrode with belt-like nanostructures which shows superaerophobicity, as shown in first and second image of Fig. 14c. Thanks to the unique array of nano-belts, the electrode exhibits extremely strong superaerophobicity: the bubble contact angle is as high as 163.5°, the average size of detached bubbles is only 5.5 μm, and the bubble adhesion force is less than 1.2 μN. Xie et al. [162] synthesized WS_2_ nanosheets by hydrothermal method (Fig. 14d). Based on the “twistronics” phenomenon of moiré superlattice, flexible WS_2_ nanobelts were obtained. The twisted bilayers active sites of the nanobelts are closer to the thermoneutral hydrogen adsorption free energy value, and the excellent superaerophobicity (with a bubble contact angle of 150.1°) reduces the release size of H_2_ bubbles, which greatly improves the catalytic performance.

#### 3D Nanostructure Arrays

The 3D nanostructures achieve cross-scale integration from nanoscale active sites to micrometer-scale mass transfer channels through multi-dimensional assembly. Their unique multi-pore system and highly dispersed functional units greatly enhance the superaerophobicity of the electrode and can even induce the bubble to bounce. The internal complex 3D structure can effectively promote the micro-convection of the electrolyte and greatly increase the mass transfer efficiency. 3D nanostructures provide an excellent reference for building high-performance superaerophobic electrodes.

**Fig. 15:**
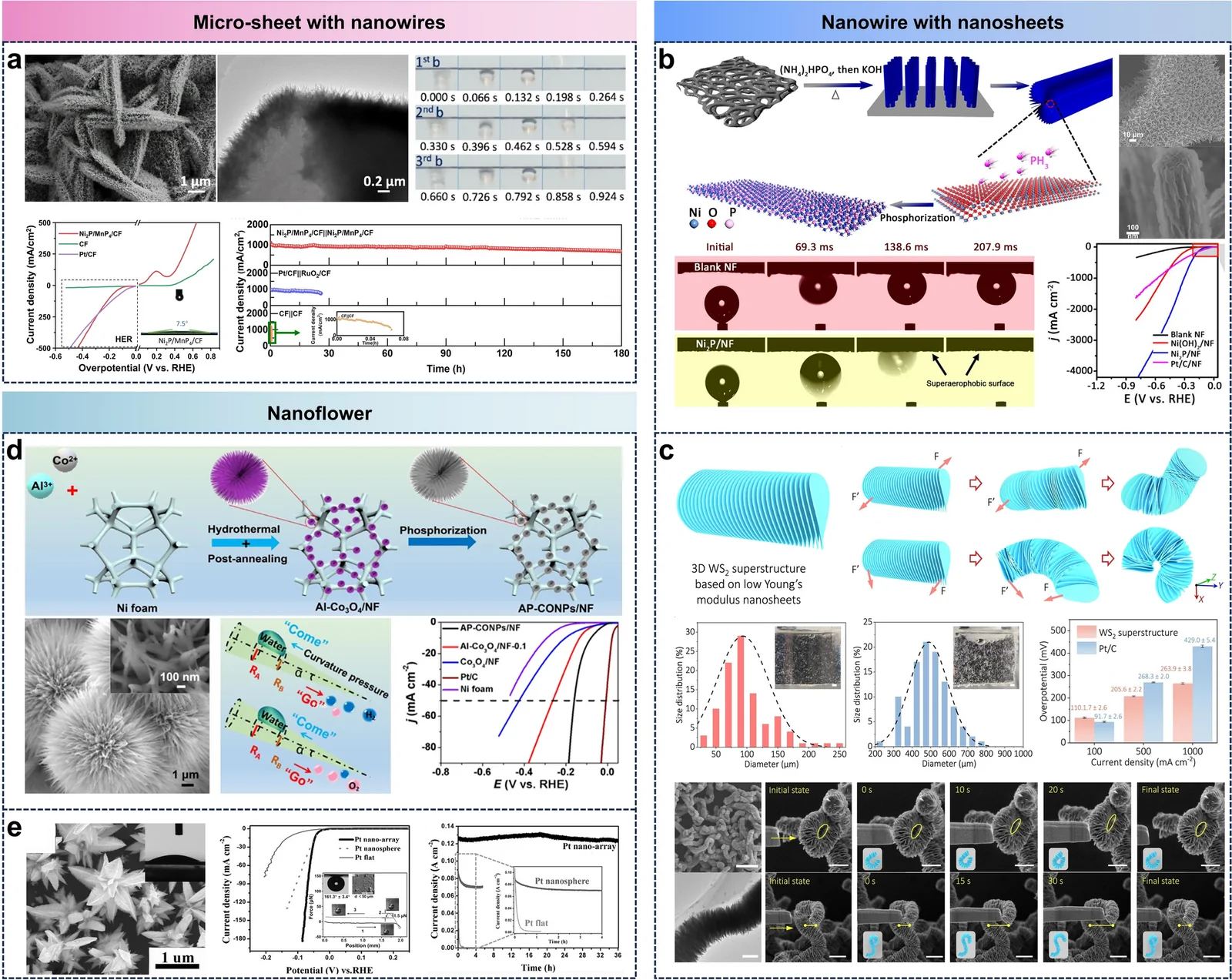
Strategies of preparing superaerophobic 3D superstructured array electrodes for HER. **a** Surface morphologies, bubbles release situation, HER and OER properties and stability of Micro-sheet with nanowire superstructured array electrode [163]. Reproduced with permission. Copyright 2024, Elsevier. **b** Preparation schematic, surface morphologies, bubbles release situation, and HER property of Nanowire with nanosheet superstructured array electrode [166]. Reproduced with permission. Copyright 2019, American Chemical Society. **c** Dynamic deformation of nanowires in shear force, bubble size distribution, HER property, and in-situ SEM images of nanowire deformation and recovery under stress [167]. Reproduced with permission. Copyright 2024, Springer Nature. **d** Preparation schematic, surface morphologies, schematic illustration of water guided by nanostructures, and HER property of nanoflower-structured array electrode [170]. Reproduced with permission. Copyright 2020, Elsevier. **e** Surface morphologies (insert is the water contact angle image), HER property (insert is the bubble adhesion curve, bubble contact angle and bubble size distribution during HER), and stability of nanoflower-structured array electrode [46]. Reproduced with permission. Copyright 2015, Wiley–VCH

Yan [163] combined hydrothermal and phosphorization strategies to construct a micro-sheet with nanowires structure on carbon fiber (Fig. 15a). This micro/nano combined 3D structure catalyst shows strong superhydrophilicity/superaerophobicity and stability, and its overpotential at high current density is much lower than that of Pt/C catalyst.

Yu et al. [166] designed a nanowire structure composed of nanosheets as shown in Fig. 15b. Compared with the blank Ni foam, the 3D nanostructured electrode exhibits superaerophobicity, the bubble can detach from the surface in just 207.9 ms. Unlike Yu’s parallel-axis nanosheets, Xie et al. [167] prepared nanowires composed of nanosheets perpendicular to the axis (Fig. 15c). This superstructure not only endows the electrode with superhydrophobicity and excellent catalytic activity, but also ensures its stability even under high current density and external force damage.

Lv et al. [170] synthesized Al–Co_3_O_4_ on Ni foam and then obtained a catalyst with a nanoflower structure through phosphorization as shown in Fig. 15d. The tips from the nanoflowers can efficiently guide the transport of water and bubbles, which effectively improves the catalytic activity. Li from Jiang’s team [46] developed a 3D nanoarray catalyst with a flower-like structure as shown in Fig. 15e. The electrode of the 3D nanoflower surface shows superaerophobicity: the bubble adhesion force is as small as 11.5 μN, the bubble contact angle is as high as 161°, and the bubble detachment diameter is less than 50 μm. In addition, the nanoflower surface of the electrode exhibit excellent HER property and stability.

Nanostructured electrodes offer abundant catalytic sites and nanoscale protrusions, promoting bubble nucleation and reducing bubble detachment size. However, the lack of macroscopic flow-guiding structures in nanostructures can lead to randomness in bubble detachment and migration behavior, increasing the probability of bubble aggregation and surface coverage. Secondly, nanostructures with high aspect ratios or weak substrate adhesion are prone to structural collapse, detachment, or morphological degradation during long-term gas evolution or under mechanical damage [175]. To address these limitations, hierarchical micro/nanostructures, oriented trenches, or columnar arrays can provide preferential bubble escape paths, thereby promoting ordered bubble transport. Furthermore, introducing aerophobic/aerophilic patterned designs [176, 177] can further modulate bubble migration trajectories. In-situ growth strategies, conductive scaffold support, and core–shell designs can significantly improve adhesion and structural durability.

### Hydrophilic Gels

In addition to the above strategies of constructing micro/nanostructures to achieve superaerophobicity, efforts have also been put to achieve superaerophobicity through other methods. The hydrophilic gel materials can effectively promote the rapid migration of hydrogen ions due to the strong hydrogen bonding ability, thereby accelerating the generation of H_2_ in the cathode reaction. In addition, their excellent flexibility and adaptability enable them to adjust the surface structure according to different application requirements, such as micro-protrusion or micro-pore design, which can not only optimize the reaction environment, so that the internal catalyst can be exposed and participate in the reaction, but also further improve the overall superaerophobicity of the electrode. At present, there are two main ways to prepare hydrophilic gel involved electrodes to achieve superaerophobicity, namely gel skeleton and gel coating. Table 3 summarized these electrodes involving hydrophilic gels and their performance parameters for hydrogen evolution.

**Table 3:** Electrochemical properties of superaerophobic electrodes

Catalyst	Electrolyte	Electrode	Aerophobic Structure	*η*_10_ (mV)	*η* at larger current density (mV@mA cm^−2^)	Stability (h@mA cm^−2^)	References
MoS_2_	0.5 M H_2_SO_4_	Cellulose nanofiber gel	Hydrophilic gel skeleton	154	192@90	100@150	[178]
DMAPA/DMAEA dual modified coatings*	1 M KOH	Ni foam	Hydrophilic gel coating	35	900@1270	60@245	[179]
BPEI/AMPS/PEGDA/HNC dual cross-linked hydrogel*	1 M KOH	Ni foam	Hydrophilic gel coating	–	938@1200	60@300	[180]
M13 virus/glutaraldehyde chemical cross-linked hydrogel*	0.5 M H_2_SO_4_	Pt electrode	Hydrophilic gel coating	146	395@40	3@250	[181]
PEI*	1 M KOH	Ni foam	Hydrophilic gel coating	–	976@1000	20@500	[182]
PAH-based hydrogels*	1 M KOH	Pt coated Ni foil	Hydrophilic gel coating	–	636@250	20@200	[183]

^*^Gel coatings instead of catalyst

**Fig. 16:**
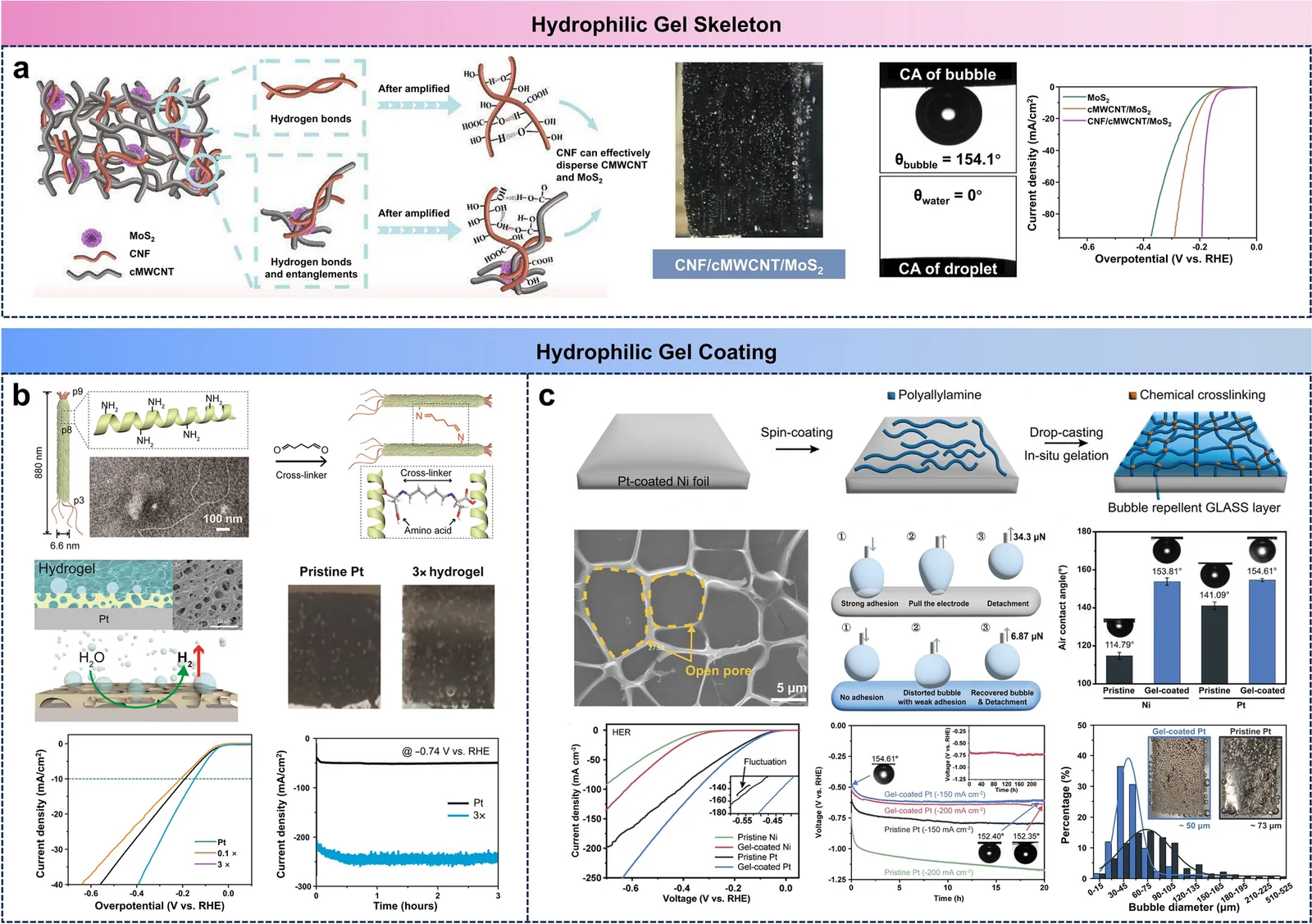
Strategies of preparing superaerophobic gel-based electrodes for HER. **a** Preparation schematic, bubble distribution, water/bubble contact angel and HER property of hydrophilic gel skeleton electrode [178]. Reproduced with permission. Copyright 2022, Elsevier. **b** Preparation schematic, HER mechanism, bubble distribution, HER property, and stability of hydrophilic gel coated electrode [181]. Reproduced with permission. Copyright 2020, American Association for the Advancement of Science. **c** Preparation schematic, surface morphologies, bubble adhesion, bubble contact angle, HER property, stability, and bubble size distribution of hydrophilic gel coated electrode [183]. Reproduced with permission. Copyright 2024, Wiley–VCH

As the name implies, the gel skeleton uses the gel as an electrode. Based on its natural conductivity or the introduction of a conductive medium, and load the catalyst on or inside the gel to prepare the gel-based electrode for HER. Chen et al. [178] prepared the catalytic electrode with a gel skeleton using cellulose nanofibers (CNFs) as the gel skeleton, carboxylated multi-walled carbon nanotubes (cMWCNTs) as the conductive medium, and hydrothermally synthesized MoS_2_ particles as the catalyst (Fig. 16a). The hydrophilic CNF gel makes the electrode superaerophobic, with a bubble contact angle as high as 154.1°. The catalyst performance of the prepared electrode is superior than that of the MoS_2_ supported by carbon cloth and the electrode without CNF skeleton with the same loading amount.

Gel coating is to coat the hydrophilic gel onto the substrate electrode to achieve superaerophobicity. Jeon et al. [181] innovatively deposited M13 bacteriophages-assembled hydrogel onto a Pt electrode. Based on the large surface area and high porosity of the hydrogel, the generated H_2_ bubbles can quickly detach from the electrode surface, thereby achieving efficient HER (Fig. 16b). From the bubble situation during the hydrogen evolution process, it can be found that the number of bubbles generated by the electrode coated with the gel with a titer of 1.5 × 10^14^ PFU mL^−1^ (where 1 × is 5.0 × 10^13^ PFU mL^−1^) is significantly more than that of the pristine Pt electrode. The Polarization curve can also prove this conclusion. Furthermore, Kang from the same team as Jeon [183] coated polyallylamine hydrochloride (PAH) and gelled it onto the Pt-coated Ni foil (Fig. 16c). The hydrophilicity of PAH hydrogel itself, coupled with the open pore structure on the surface, makes the aerophobicity of the electrode coated with PAH hydrogel significantly better than that of the pristine electrode. The bubble adhesion force is only 6.87 μN, and the bubble contact angle is as high as 154.61°. Furthermore, during the HER process, the gel-coated electrode exhibits excellent catalytic activity and stability, with the average diameter of bubbles detaching from electrode surface being only ~ 50 μm.

Hydrophilic gels exhibit ultralow bubble adhesion and excellent gas-slip properties. However, their practical durability is constrained by uncontrollable thickness, swelling-induced delamination, and long-term electrochemical degradation. Developing micro-/nano-scale ultrathin gel coating technologies and chemically stable hydrogels are crucial.

### Comparison of Electrode Structures for Bubble Regulation

Microstructure arrays, nanostructure arrays, and hydrophilic gel coatings have all been shown to effectively promote bubble release, but their regulatory mechanisms are governed by different length scales and interfacial physicochemical principles. Thus, it is crucial to construct comparisons to elucidate how different electrode structures modulate bubble dynamics and thus affect the performance of HER.

Microstructure arrays primarily guide the detachment of bubbles through directional gas channels, thereby achieving efficient bubble release, but the improvement in surface area is limited. In contrast, nanostructure arrays, with their abundant nanoscale protrusions and active sites, effectively improve gas expulsion and catalytic activity. However, the stability of nanostructures under high current density and long-term application still warrants further investigation. Distinct from solid structural modulation, hydrophilic gel coatings regulate gas evolution behavior through interfacial wettability and hydration-layer effects, facilitating spontaneous detachment. Additionally, continuous water channels within gels facilitate electrolyte transport and suppress gas film formation. Despite these merits, hydrogel layers may introduce additional charge-transfer resistance and mass transport barriers, particularly when thickness and crosslinking density are not optimized [184].

From a fabrication perspective, these structures also present different challenges. For microstructure arrays, precise geometric control is crucial, as the spacing and height of the structures directly affect the escape path of bubbles. Nanostructure arrays, on the other hand, need to avoid structural collapse after long-term service. For hydrophilic gels, achieving uniform coating thickness and strong interfacial adhesion remains a challenge.

Overall, microstructures favor directional transport, nanostructures maximize active area and nucleation, while hydrophilic gels minimize interfacial adhesion. Integrating these advantages through hierarchical or hybrid designs represents a promising pathway for simultaneously optimizing bubble regulation and HER performance.

## Challenge and Outlook

This review systematically summarizes the significant progress in achieving precise manipulation of gas bubbles in hydrogen evolution reaction electrodes through the rational design of micro/nano-structured surfaces. By delving into the principles of interface wetting engineering, we have illustrated how superaerophobic electrodes effectively optimize key performance parameters, including overpotential reduction, bubble dynamics, mass transfer efficiency, and catalyst stability. Furthermore, strategic design principles for constructing high-performance superaerophobic electrodes, based on tailoring surface morphology and structure, are comprehensively discussed. This review aims to provide robust theoretical guidance and practical insights for researchers and engineers working in this field.

In the HER process, the underwater superaerophobic electrode has become one of the key strategies in improving electrolysis efficiency by minimizing the adhesion and coverage of bubbles on the electrode surface. Despite significant achievements, several key challenges remain to bridge the gap between laboratory research and widespread industrial applications. Based on current research progress and challenges, future development trends in this field will focus on the following aspects: (1) Multiscale bubble dynamics theory and accurate modeling. Future research will focus on constructing a multi-scale theoretical model covering the entire process from nanobubble nucleation to macroscopic bubble desorption. This requires not only refining traditional force balance models (such as incorporating Marangoni forces and electric fields), but also leveraging advanced in-situ characterization techniques such as surface plasmon resonance (SPR) and machine learning methods to accurately predict and regulate bubble evolution. (2) Exploring new materials that combine high conductivity and high stability. Developing novel electrode materials is fundamental to improving overall performance. Future research should focus on significantly improving catalytic efficiency in industrial production through electrodes with high conductivity and high stability. (3) Design intelligent adaptive and dynamic response electrode structures. Simple superaerophobic electrode is no longer sufficient to meet the demands of complex operating conditions. Research exemplified by aerophilic/aerophobic patterned electrodes has successfully achieved the directional guidance of bubbles, but the next generation of electrode design will evolve toward dynamically adaptive intelligent systems. These adaptive structures can not only manage bubbles efficiently, but also have excellent mechanical elasticity, achieving a leap from “static structural design” to “dynamic functional regulation.” (4) Active and precise control of bubble behavior. Future advanced bubble management will move beyond passive “rapid desorption” to proactive intervention and programmed control of bubble behavior. This requires the integration of multidisciplinary approaches: based on existing nanobubble “seed” strategies, external fields such as magnetic and electric fields can be introduced to actively manipulate the dynamics of bubbles, achieving more efficient bubble transport and removal. (5) Artificial intelligence (AI) and deep learning guide electrode development process. A dedicated materials database was built, and machine learning algorithms were used to reverse engineer novel electrode materials, significantly shortening the R&D cycle. Simultaneously, the AI model integrates real-time multimodal data, including current, temperature, solution environment, and bubble images, to predict and warn of bubble behavior and dynamically optimize the electrolier’s operating parameters, ultimately achieving global intelligent optimization from materials design to system operation. (6) Future research should focus on addressing the challenges of large-scale production and industrial application of superhydrophobic electrodes. Currently, most progress remains in the laboratory stage, and micro/nano fabrication faces numerous challenges in terms of cost, yield, and equipment requirements. Developing low-cost, large-area fabrication strategies while ensuring their operational stability in industrial electrolyzers is crucial for bridging laboratory innovation and practical H_2_ production. (7) Interdisciplinary technical collaboration in bubble management. The evolution of bubbles has a wide-ranging impact on processes such as photocatalytic gas production, thermal-assisted electrolysis, and photovoltaic-electrolysis systems. The superaerophobic interface has universal advantages in alleviating gas accumulation and optimizing bubble transport. Future research should focus on adapting interface wetting engineering to various electrochemical environments, thereby achieving enhanced collaborative performance in various energy technologies for gas generation.

In general, the future development of underwater superhydrophobic electrodes will be characterized by more refined theoretical models, more intelligent electrode designs, more composite material systems, more precise bubble control, and more intelligent technological applications. Breakthroughs in these areas will strongly promote the large-scale industrial application of efficient, stable, and low-cost green hydrogen production technologies.
